# Identification of Daphnane Diterpenoids from Flower Buds and Blooming Flowers of *Daphne odora* Using UHPLC-Q-Exactive-Orbitrap MS

**DOI:** 10.3390/plants14172616

**Published:** 2025-08-22

**Authors:** Kouharu Otsuki, Kousei Miyamoto, Mami Goto, Mi Zhang, Takashi Kikuchi, Wei Li

**Affiliations:** Faculty of Pharmaceutical Sciences, Toho University, Miyama 2-2-1, Funabashi 274-8510, Chiba, Japanzhangmi495@gmail.com (M.Z.); takashi.kikuchi@phar.toho-u.ac.jp (T.K.)

**Keywords:** *Daphne odora*, diterpenoid, flower, Thymelaeaceae, LC-MS

## Abstract

*Daphne odora* is an evergreen shrub belonging to the Thymelaeaceae family that is widely cultivated as an ornamental garden plant. Its roots, leaves, and flowers have traditionally been used in Chinese medicine to treat pain, skin diseases, and rheumatism. While previous phytochemical studies have reported the presence of phenols, coumarins, biflavonoids, lignans, and daphnane diterpenoids in *D. odora*, its flowers remain largely unexplored. In the present study, the first comprehensive investigation of daphnane diterpenoids contained in the flower buds and blooming flowers of *D. odora* was conducted using ultra-high-performance liquid chromatography coupled with Q-Exactive-Orbitrap high-resolution mass spectrometry (UHPLC-Q-Exactive-Orbitrap MS). A total of 30 daphnane diterpenoids were identified, including 12 previously unreported compounds, through detailed analysis of their retention times and MS/MS fragmentation patterns. Comparative profiling revealed that flower buds contained a higher abundance and greater diversity of daphnane diterpenoids than flowers. Furthermore, LC–MS-guided isolation enabled the purification of a novel compound, daphneodorin I (**16**), and its structure was elucidated through extensive physicochemical and spectroscopic analyses. Compound **16** represents the first daphnane diterpenoid with a *Z*-configured phenolic acyl moiety isolated from plants of the Thymelaeaceae family.

## 1. Introduction

*Daphne odora* Thunb. is an evergreen shrub belonging to the Thymelaeaceae family. Its roots, leaves, and flowers have been traditionally used in Chinese medicine for the treatment of pain, skin disorders, and rheumatism [1,2]. Today, this plant is widely cultivated as an ornamental species worldwide and is commonly known as “winter daphne” because of its fragrant pinkish-white flowers that bloom in early spring. Previous phytochemical studies on *D. odora* have reported the isolation of phenols, coumarins, biflavonoids, lignans, and daphnane diterpenoids [3,4,5,6,7,8,9]. Furthermore, daphnane diterpenoids were isolated from the leaves and stems of *D. odora* in our previous study [10,11]. Daphnane diterpenoids are a characteristic class of diterpenoids found in plants of the Thymelaeaceae family [12]. Their chemical structures are highly diverse, featuring a *trans*-fused 5/7/6 tricyclic ring system with various oxygenated functional groups. These diterpenoids have been extensively studied for their wide range of biological activities, including anticancer, anti-HIV, and analgesic effects, and are considered potential lead compounds for drug development. In particular, structural variations, such as those in the orthoester moiety and acyl substituents, have been reported to markedly influence both bioactivity and toxicity [13,14,15]. Therefore, chemical investigation of daphnane diterpenoids present in plants is essential to understand their pharmacological potential and safety profiles.

**Figure 1:**
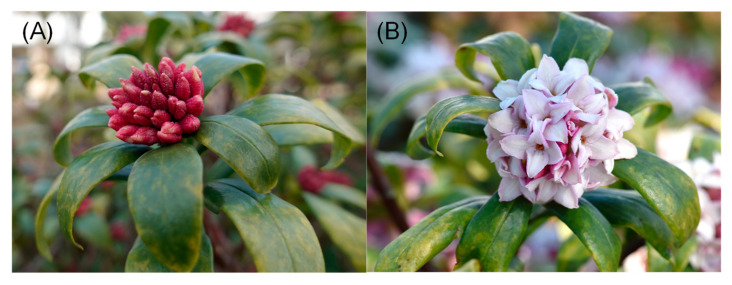
Flower buds before blooming (**A**) and fully opened flowers (**B**) of *D. odora*.

The flowers of *D. odora* progress through several distinct stages, beginning with the swelling of pink buds in late winter, which then open into clusters of fragrant, four-lobed flowers, typically pink or white (Figure 1) [1]. Moreover, floral secondary metabolite profiles vary significantly across different developmental stages [16,17]. Although the flowers of *D. odora* have been used in traditional Chinese medicines, their chemical constituents remain largely unexplored. *D. genkwa*, a medicinal plant in the same genus *Daphne*, has yielded numerous daphnane diterpenoids from its flower buds [18,19]. Inspired by these findings, the present study focused on the daphnane diterpenoids present in the flower buds and blooming flowers of *D. odora*. Herein, we report the identification of daphnane diterpenoids in the flowers of *D. odora* using an ultra-high-performance liquid chromatography coupled with high-resolution mass spectrometry (UHPLC-Q-Exactive-Orbitrap MS), as well as LC-MS-guided isolation and structural elucidation of a previously undescribed daphnane diterpenoid (**16**).

## 2. Results and Discussion

### 2.1. Detection of Daphnane Diterpenoids in the Flower Buds and Flowers of D. odora Using LC-MS/MS

**Figure 2:**
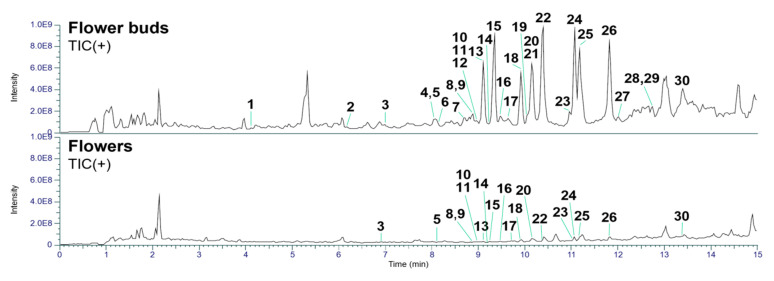
Total ion chromatograms in positive ion mode of the flower buds and flowers of *D. odora*.

In the present study, we separately collected flower buds prior to blooming and fully opened flowers for LC-MS analysis. The collected samples were extracted with MeOH and subsequently partitioned between EtOAc and H_2_O. The EtOAc-soluble fractions were further subjected to pretreatment using a Sep-Pak tC_18_ Plus Long cartridge and analyzed by UHPLC-Q-Exactive-Orbitrap MS. The acquired raw data were preprocessed using MZmine 2 software [20] and subsequently analyzed with the in-house developed CNPs-MFSA application [21] for the recognition of daphnane diterpenoids. The above qualitative analysis revealed that the flower buds contained a higher abundance of daphnane diterpenoids and more previously undescribed compounds than the flowers (Figure 2 and Table S1). Furthermore, detailed structural identification of the detected daphnane diterpenoids was performed following the same protocol described in our previous studies [22]. As summarized in Table 1, several daphnane diterpenoids were commonly identified in both flowers and buds, with 19 compounds detected in the flowers and 30 compounds detected in the buds.

The identified daphnane diterpenoids were classified as two types, daphnane orthoesters (**1**–**4**, **6**–**8**, **10**–**20**, and **22**–**30**) and macrocyclic daphnane orthoesters (**5**, **9**, and **21**), based on their chemical structural characteristics [12]. Moreover, several previously unreported daphnane diterpenoids (**6**–**8**, **10**, **12**, **14**, **16**, **17**, **19**, **23**, **28**, and **30**) have been identified in the buds of *D. odora*. Their structural elucidation based on MS/MS fragmentation analysis is described below.

**Table 1:** Tentative identification of daphnane diterpenoids in the flower buds and flowers of *D. odora*.

No.	t*_R_*, min (+/−)	Precursor Ion, *m*/*z* (Error, ppm)		Molecular Formula	Tentative Identification	Reference
		[M + H]^+ a^	[M–HCOO]^− b^			
**1**	4.21/4.19	487.2325 (−0.33)	485.2189 (1.74)	C_27_H_34_O_8_	3-deoxo-1,2-dihydro-3-hydroxydaphnetoxin	[23,24]
**2**	6.09/6.08	543.2587 (−0.24)	587.2507 (1.51)	C_30_H_38_O_9_	peddiea factor A_1_	[25]
**3**	6.93/6.95	545.2746 (0.16)	589.2662 (1.25)	C_30_H_40_O_9_	12-hydroxyexcoecariatoxin	[26]
**4**	8.10/8.08	585.2686 (−1.38)	629.2609 (0.91)	C_32_H_40_O_10_	peddiea factor V_2_	[25]
**5** ^c^	8.10/8.11	1028.4264 (−1.02)	1055.3934 (1.53)	C_55_H_62_O_18_	daphneodrin B	[10]
**6** ^e^	8.13/8.14	705.2899 (−0.90)	703.2770 (1.39)	C_39_H_44_O_12_	12-*O*-(*E*)-caffeoyl-9,13,14-ortho-(2*E*,4*E*,6*E*)-decatrienylidyne-5*β*,12*β*-dihydroxyresiniferonol-6*α*,7*α*-oxide	-
**7** ^e^	8.68/8.70	689.2951 (−0.77)	687.2814 (0.42)	C_39_H_44_O_11_	12-*O*-(*Z*)-coumaroyl-9,13,14-ortho-(2*E*,4*E*,6*E*)-decatrienylidyne-5*β*,12*β*-dihydroxyresiniferonol-6*α*,7*α*-oxide	-
**8** ^e^	8.88/8.87	719.3056 (−0.83)	717.2920 (0.48)	C_40_H_46_O_12_	12-*O*-(*Z*)-feruloyl-9,13,14-ortho-(2*E*,4*E*,6*E*)-decatrienylidyne-5*β*,12*β*-dihydroxyresiniferonol-6*α*,7*α*-oxide	-
**9** ^c^	8.88/8.89	970.4219 (−0.09)	997.3874 (1.05)	C_53_H_60_O_16_	daphneodorin A	[10]
**10** ^e^	8.93/8.95	689.2952 (−0.60)	687.2819 (1.13)	C_39_H_44_O_11_	12-*O*-(*E*)-coumaroyl-9,13,14-ortho-(2*E*,4*E*,6*Z*)-decatrienylidyne-5*β*,12*β*-dihydroxyresiniferonol-6*α*,7*α*-oxide	-
**11**	8.97/8.95	587.2850 (−0.17)	631.2769 (1.36)	C_32_H_42_O_10_	yuanhuadin	[27]
**12** ^e^	8.97/8.95	707.3051 (−1.63)	705.2924 (1.09)	C_39_H_46_O_12_	12-*O*-(*E*)-caffeoyl-9,13,14-ortho-(2*E*,4*E*)-decadienylidyne-5*β*,12*β*-dihydroxyresiniferonol-6*α*,7*α*-oxide	-
**13** ^c^	9.10/9.12	689.2953 (−0.42)	687.2814 (0.42)	C_39_H_44_O_11_	daphneodorin E	[11]
**14** ^e^	9.18/9.17	719.3061 (−0.16)	763.2982 (1.35)	C_40_H_46_O_12_	12-*O*-(*E*)-feruloyl-9,13,14-ortho-(2*E*,4*E*,6*Z*)-decatrienylidyne-5*β*,12*β*-dihydroxyresiniferonol-6*α*,7*α*-oxide	-
**15** ^c^	9.35/9.34	719.3051 (−1.60)	763.2970 (−0.17)	C_40_H_46_O_12_	actilobin C	[28]
**16** ^e,f^	9.47/9.49	691.3111 (−0.28)	689.2972 (0.65)	C_39_H_46_O_11_	12-*O*-(*Z*)-coumaroyl-9,13,14-ortho-(2*E*,4*E*)-decadienylidyne-5*β*,12*β*-dihydroxyresiniferonol-6*α*,7*α*-oxide	-
**17** ^e^	9.67/9.68	721.3220 (0.15)	719.3081 (1.12)	C_40_H_48_O_12_	12-*O*-(*Z*)-feruloyl-9,13,14-ortho-(2*E*,4*E*)-decadienylidyne-5*β*,12*β*-dihydroxyresiniferonol-6*α*,7*α*-oxide	-
**18** ^c^	9.91/9.92	691.3109 (−0.54)	689.2968 (0.11)	C_39_H_46_O_11_	daphneodorin D	[11]
**19** ^d^	10.05/10.03	649.3000 (−1.04)	693.2921 (0.58)	C_37_H_44_O_10_	1,2-dihydroyuanhuajine	[24]
**20** ^c^	10.15/10.16	721.3212 (−0.95)	765.3127 (−0.05)	C_40_H_48_O_12_	actilobin D	[28]
**21** ^c^	10.15/10.16	775.3693 (0.67)	819.3587 (−1.25)	C_44_H_54_O_12_	gnidimacrin	[29]
**22** ^c^	10.36/10.35	647.2845 (−0.91)	691.2759 (−0.09)	C_37_H_42_O_10_	yuanhuajine	[7]
**23** ^e^	10.93/10.91	673.3004 (−0.46)	717.2928 (1.58)	C_39_H_44_O_10_	12-*O*-(*E*)-cinnamoyl-9,13,14-ortho-(2*E*,4*E*,6*Z*)-decatrienylidyne-5*β*,12*β*-dihydroxyresiniferonol-6*α*,7*α*-oxide	-
**24** ^c^	11.07/11.06	673.2999 (−1.27)	717.2918 (0.22)	C_39_H_44_O_10_	12-*O*-(*E*)-cinnamoyl-9,13,14-ortho-(2*E*,4*E*,6*E*)-decatrienylidyne-5*β*,12*β*-dihydroxyresiniferonol-6*α*,7*α*-oxide	[7]
**25** ^c^	11.18/11.16	649.2998 (−1.42)	693.2917 (0.14)	C_37_H_44_O_10_	yuanhuacine/odoracin	[30]
**26** ^c^	11.81/11.82	675.3157 (−0.95)	719.3074 (0.10)	C_39_H_46_O_10_	12-*O*-(*E*)-cinnamoyl-9,13,14-ortho-(2*E*,4*E*)-decadienylidyne-5*β*,12*β*-dihydroxyresiniferonol-6*α*,7*α*-oxide	[7]
**27**	12.01/12.00	635.3215 (0.02)	679.3135 (1.70)	C_37_H_46_O_9_	actilobin F	[29]
**28** ^e^	12.74/12.73	691.3474 (−0.38)	735.3394 (1.03)	C_40_H_50_O_10_	12-*O*-(2*E*,4*E*,6*E*)-decatrienoyl-9,13,14-ortho-(2*E*,4*E*,6*E*)-decatrienylidyne-5*β*,12*β*-dihydroxyresiniferonol-6*α*,7*α*-oxide	-
**29**	12.77/12.76	637.3369 (−0.31)	681.3286 (0.76)	C_37_H_48_O_9_	wikstroemia factor M1	[27]
**30** ^d^	13.39/13.38	693.3630 (−0.50)	737.3547 (0.66)	C_40_H_52_O_10_	12-*O*-(2*E*,4*E*,6*E*)-decatrienoyl-9,13,14-ortho-(2*E*,4*E*)-decadienylidyne-5*β*,12*β*-dihydroxyresiniferonol-6*α*,7*α*-oxide	[24]

^a^ Detected as [M + NH4]^+^ ion for compounds **5** and **9**. ^b^ Detected as [M–H]^−^ ion for compounds **1**, **6**–**8**, **10**, **12**, **13**, and **16**–**18**. ^c^ Identifications were confirmed by comparison of retention times and product ion spectra with authentic standards. ^d^ Compounds previously identified in other Daphne species using LC-MS analysis. ^e^ Previously unreported compounds. ^f^ Compounds were isolated in the present study.

### 2.2. Identification of Previously Unreported Daphnane Diterpenoids by MS/MS Fragmentation Analysis

**Figure 3:**
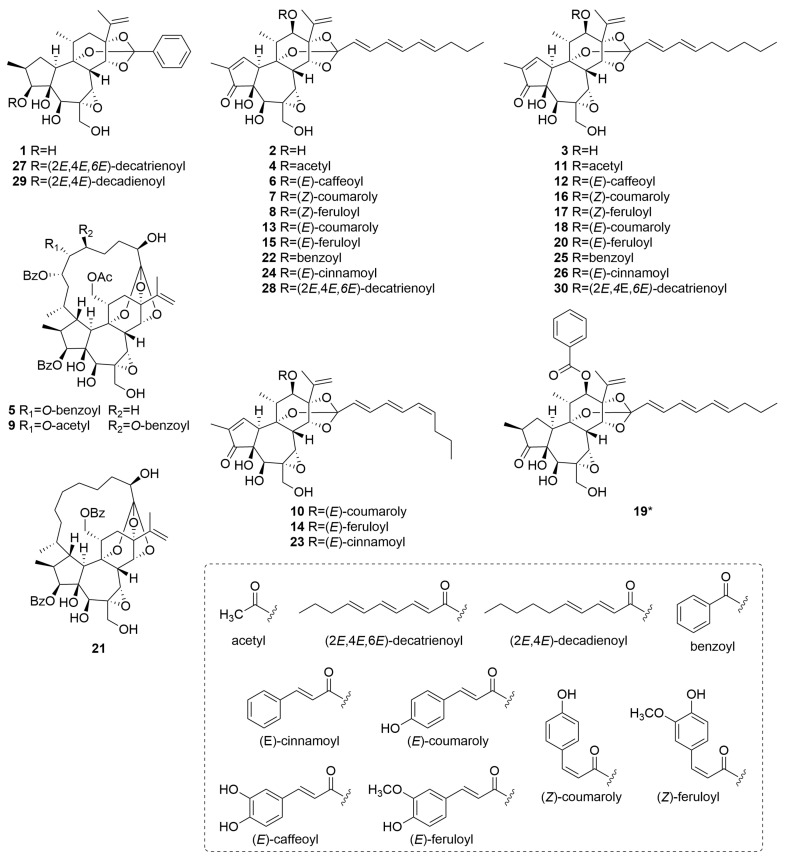
Structures of daphnane diterpenoids **1**–**30** identified in *D. odora*. Asterisks indicate previously unreported compounds.

Compounds **6**, **12**, and **28** exhibited a series of characteristic product ions in the positive ion mode at *m*/*z* 359 (C_20_H_23_O_6_^+^) → *m*/*z* 341 (C_20_H_21_O_5_^+^) → *m*/*z* 269 (C_17_H_17_O_3_^+^), indicating that they share a typical daphnane skeleton bearing a 1,2-en-3-oxo-4,5-dihydroxy-6,7-epoxy moiety with a substituent at C-12 (Figures S1–S3). In the negative ion mode, the observation of [M−RCOOH−CH_2_O−H]^−^ ions is useful for identifying the acyl moiety attached via orthoester linkages on the C-ring [22]. For compounds **6** and **28**, a neutral loss of C_10_H_14_O_2_ from the deprotonated molecular ion suggested the presence of a 2,4,6-decatrienoyl moiety linked to the C-ring through an orthoester bond. In contrast, compound **12** exhibited a neutral loss of C_10_H_16_O_2_, indicating the presence of a 2,4-decadienoyl moiety at the same position. Structural information on the acyl moiety at C-12 was further obtained by analyzing product ions below *m*/*z* 250. Compounds **6** and **12** exhibited common characteristic ions corresponding to C_9_H_7_O_3_^+^ and C_9_H_7_O_4_^−^. Given that numerous daphnane diterpenoids isolated from *D. odora* possess phenylpropanoid-derived substituents, such as cinnamoyl, coumaroyl, and feruloyl groups biosynthesized from phenylalanine, these diagnostic ions were attributed to the caffeoyl group [11]. Therefore, the substituent at C-12 in compounds **6** and **12** was assigned as a caffeoyl group. Compound **28** showed only diagnostic ions corresponding to C_10_H_13_O^+^, C_7_H_7_O^+^, and C_10_H_13_O_2_^−^, indicating that a 2,4,6-decatrienoyl moiety was attached to C-12. Thus, these findings demonstrate that compounds **6**, **12**, and **28** are previously unreported compounds. Their proposed structures are illustrated in Figure 3.

Based on the product ion spectra obtained in both positive and negative ion modes, compounds **7**, **8**, **10**, **14**, **16**, **17**, and **23** were suggested to possess a daphnane skeleton with a substituent at C-12. Detailed comparisons of the retention behavior, predicted molecular formulas, and MS/MS fragmentation patterns with those of authentic standards revealed that these compounds are novel geometric isomers of the known compounds **13**, **15**, **18**, **20**, and **24**, respectively (Figure S4). Specifically, compounds **7** (t*_R_* = 8.68 min) and **10** (t*_R_* = 8.93 min) correspond to isomers of daphneodorin E (**13**, t*_R_* = 9.10 min) (Figures S5 and S6); compounds **8** (t*_R_* = 8.88 min) and **14** (t*_R_* = 9.18 min) to actilobin C (**15**, t*_R_* = 9.35 min) (Figures S7 and S8); compound **16** (t*_R_* = 9.47 min) to daphneodorin D (**18**, t*_R_* = 9.91 min) (Figure S9); compound **17** (t*_R_* = 9.67 min) to actilobin D (**20**, t*_R_* = 10.15 min) (Figure S10); and compound **23** (t*_R_* = 10.93 min) to 12-*O*-(*E*)-cinnamoyl-9,13,14-ortho-(2*E*,4*E*,6*E*)-decatrienylidyne-5*β*,12*β*-dihydroxyresiniferonol-6*α*,7*α*-oxide (**24**, t*_R_* = 11.07 min) (Figure S11). These geometric isomers are presumed to arise from differences in the *E*/*Z* configuration of double bonds present in either the aliphatic or phenolic acyl moieties. Our previous study demonstrated that, under consistent LC conditions, isomers bearing a (2*E*,4*E*,6*Z*)-decatrienoyl moiety eluted 0.15 min earlier than their (2*E*,4*E*,6*Z*) counterparts [24]. Based on this finding, compounds **10**, **14**, and **23**, which eluted approximately 0.15 min earlier than compounds **13**, **15**, and **24**, respectively, were suggested to possess a (2*E*,4*E*,6*Z*)-decatrienoyl moiety attached to the C-ring via an orthoester linkage.

In contrast to the previously discussed isomers, compounds **7**, **8**, **16**, and **17** eluted approximately 0.45 min earlier than their known analogs, a more pronounced shift that is likely attributable to the presence of a Z-configured double bond in the aromatic acyl substituent. Specifically, compounds **7** and **16** were presumed to possess a (*Z*)-coumaroyl moiety at C-12, whereas compounds **8** and **17** were suggested to possess a (*Z*)-feruloyl moiety at C-12. Nevertheless, the structural assignment of these compounds could not be conclusively determined from MS/MS fragmentation and retention time data alone. To clarify their structures, LC–MS-guided isolation was performed on the flower buds, leading to the successful purification of compound **16** for detailed spectroscopic analysis.

### 2.3. LC-MS Guided Isolation and Structural Elucidation of Compound **16**

**Figure 4:**
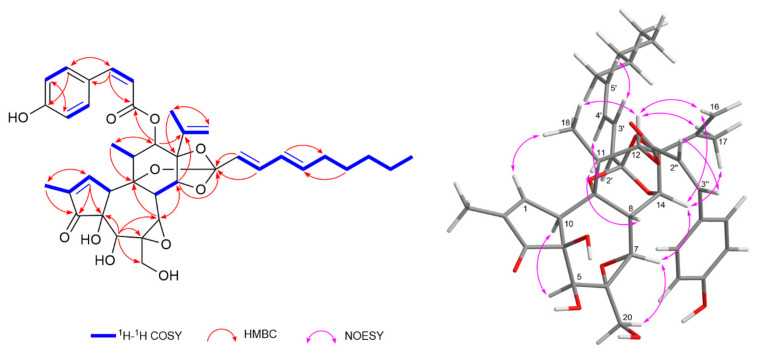
Key ^1^H–^1^H COSY, HMBC, and NOESY correlations for compound **16**.

Compound **16** was isolated as a colorless solid, [α]^23^_D_ + 16.4 (c 0.07, MeOH). Its molecular formula was determined as C_39_H_46_O_11_ from the negative HRESIMS data, showing a deprotonated molecular ion at *m*/*z* 689.2967 [M−H]^−^ (calcd for C_39_H_45_O_11_, 689.2967). The ^1^H- and ^13^C-NMR resonances of compound **16** were virtually identical to those of daphneodorin D (**18**), except for resonances assignable to the phenolic acyl moiety at C-12 (Table 2). A detailed analysis revealed characteristic resonances for a daphnane orthoester, including an isopropenyl moiety at *δ*_H_ 4.74 (Ha-16), 4.92 (Hb-16), 1.69 (H_3_-17), *δ*_C_ 142.7 (C-15), 113.7 (C-16), and 18.8 (C-18); an *α*,*β*-unsaturated carbonyl group at *δ*_H_ 7.57 (H-1), *δ*_C_ 161.3 (C-1), 136.6 (C-2), and 209.6 (C-3); an epoxy group at *δ*_H_ 3.23 (H-7), *δ*_C_ 58.6 (C-6), and 66.3 (C-7); as well as a quaternary carbon at *δ*_C_ 116.6 (C-1′) consistent with the orthoester group. The presence of a 2,4-decadienylidyne moiety was indicated by the proton resonances for a conjugated diene at *δ*_H_ 5.56 (d, *J* = 15.4 Hz, H-2′), 6.57 (dd, *J* = 15.4, 10.9 Hz, H-3′), 5.99 (dd, *J* = 15.2, 10.9 Hz, H-4′), and 5.80 (dt, *J* = 15.2, 7.2 Hz, H-5′), a *n*-pentyl moiety with four methylenes at *δ*_H_ 2.06 (H-6′), 1.35 (H-7′), 1.25 (H-8′), and 1.28 (H-9′), and a terminal methyl group at *δ*_H_ 0.86 (t, *J* = 7.0 Hz, H_3_-10′). The large coupling constants between H-2′/H-3′ and H-4′/H-5′ (*J* = 15.4 and 15.2 Hz, respectively) and the NOESY correlations between H-2′/H-4′ and H-3′/H-5′ indicated a 2*E*,4*E* geometry of the conjugated diene (Figure 4). The position of the decadienylidyne moiety was confirmed from the HMBC correlations from H-14, H-2′, and H-3′ to C-1′. Furthermore, compound **16** exhibited proton resonances consistent with a *p*-coumaroyl moiety, including olefinic protons at *δ*_H_ 5.78 (d, *J* = 12.2 Hz, H-2″) and 7.05 (d, *J* = 12.2 Hz, H-3″), as well as aromatic protons at *δ*_H_ 6.89 (d, *J* = 8.4 Hz, H-6″,8″) and 7.15 (d, *J* = 8.4 Hz, H-5″,9″). The relatively small coupling constant between H-2″ and H-3″ (*J* = 12.2 Hz) indicated a *Z*-configured *p*-coumaroyl moiety. The HMBC correlation from H-12 to the ester carbonyl carbon at *δ*_C_ 165.1 (C-1″) confirmed that the (*Z*)-coumaroyl moiety was attached at C-12 (Figure 4). Thus, the structure of compound **16** was determined as 12-*O*-(*Z*)-coumaroyl-9,13,14-ortho-(2*E*,4*E*)-decadienylidyne-5*β*,12*β*-dihydroxyresiniferonol-6*α*,7*α*-oxide, a geometric isomer of daphneodorin D (**18**), and was named daphneodorin I.

## 3. Materials and Methods

### 3.1. General Experimental Producers

Optical rotation, UV, ECD, IR, NMR, and HRESI-MS measurements were performed using the instruments and conditions described in the literature [31]. Column chromatography was carried out on Diaion HP-20 (Mitsubishi Chemical Corporation, Tokyo, Japan), as well as ODS silica gel, silica gel, and preparative HPLC, following the procedures described in the literature [31].

### 3.2. Plant Materials

Plants of *D. odora* were cultivated at the Toho University Medicinal Plant Garden in Chiba, Japan. Blooming flowers were collected in late February 2020 and flower buds were collected in early February 2021. The plant materials were identified by one of the authors (W.L.). Voucher specimens THMPG-7 (blooming flowers) and THMPG-8 (flower buds) were deposited at the Department of Pharmacognosy, Faculty of Pharmaceutical Sciences, Toho University, Japan.

### 3.3. Extraction and Isolation

Air-dried flower buds (756 g) and flowers (956 g) of *D. odora* were extracted four times with methanol (MeOH, 5 L for flower buds and 8 L for flowers, 1 h each) using ultrasonic extraction at room temperature. The resulting extracts were filtered and concentrated under reduced pressure at a temperature not exceeding 40 °C. The MeOH extracts of the flower buds (515 g) and flowers (434 g) were each suspended in H_2_O and partitioned with ethyl acetate (EtOAc). The EtOAc fractions of the flower buds (55.1 g) and flowers (64.3 g) were collected for LC-MS analysis and subsequent isolation.

For the isolation process, the EtOAc fraction of the flower buds (55.1 g) was subjected to Diaion HP-20 column chromatography and eluted with a gradient of MeOH/H_2_O (5:5 to 10:0, *v*/*v*) to yield four fractions (E1 to E4). Fraction E3 (8.39 g) was further separated by ODS column chromatography using a stepwise gradient of MeOH−H_2_O (from 7:3 to 10:0, *v*/*v*), affording 13 fractions (E3-1 to E3-13). Combined fractions E3-6 and E3-7 (2.28 g) were subjected to silica gel column chromatography and eluted with EtOAc−MeOH (1:1, *v*/*v*) followed by MeOH−HCOOH (95:5, *v*/*v*) to give two subfractions (E3-6-1 and E3-6-2). Finally, fraction E3-6-1 (754 mg) was repeatedly purified by RP-HPLC (70% CH_3_CN) to afford compound **16** (0.7 mg).

#### Daphneodorin I (**16**)

Colorless solid; [α]^23^_D_ +16.4 (*c* 0.07, MeOH); UV (MeOH) *λ*_max_ nm (log *ε*) 230 (4.68), 314 (4.24); ECD (MeOH) [*θ*]^25^ (nm): 17,222 (206), −55,825 (228), 21,744 (246), −11,284 (309), 4892 (351); IR (KBr) cm^−1^: 3429, 2955, 2927, 2856, 1703, 1631, 1605, 1514, 1456, 1381, 1363, 1312, 1281, 1153, 1109, 1085, 1057, 1040, 1010; ^1^H- and ^13^C-NMR spectroscopic data, see Table 2; HRESI-MS (negative) *m*/*z*: 689.2967 [M−H]^−^ (calcd for C_39_H_45_O_11_: 689.2967).

### 3.4. Qualitative Analysis of Flower Buds and Flower of D. odora

#### 3.4.1. Sample Preparation

The EtOAc fractions of the flower buds and flowers (10.0 mg each) were dissolved in 50% MeOH (1.0 mL) and loaded onto a conditioned Sep-Pak tC18 Plus Long Cartridge (Waters, Milford, MA, USA), washed with 50% MeOH (10 mL), and subsequently eluted with 100% MeOH (10 mL). Finally, the 100% MeOH eluates were then filtered through a 0.20 mm membrane filter (Merck Millipore, Burlington, MA, USA) prior to qualitative analysis.

#### 3.4.2. LC-MS/MS Conditions

LC–MS/MS analysis was performed using an ultra-high-performance liquid chromatography coupled with high-resolution mass spectrometry (UHPLC-Q-Exactive-Orbitrap MS, Thermo FisherScientific, Waltham, MA, USA) equipped with a heated electrospray ionization (HESI) source, under conditions consistent with a previous study [23]. Chromatographic separation was carried out on a YMC-Triart C_18_ column (150 mm × 2.1 mm, 1.9 μm) at a flow rate of 0.4 mL/min with the column oven maintained at 40 °C. The mobile phase consisted of 0.1% formic acid in water (A) and 0.1% formic acid in acetonitrile (B), using a linear gradient from 50% to 100% B over 15 min. Mass spectrometric data were acquired in profile mode over an *m*/*z* range of 300–1500, with a resolution of 35,000 (*m*/*z* 200) in full MS mode and 17,500 (*m*/*z* 200) in the dd-MS^2^ mode. Fragmentation was carried out using higher-energy collisional dissociation (HCD) with a normalized collision energy of 15–20 eV in positive ion mode and 10 eV in negative ion mode [24].

#### 3.4.3. Data Analysis

All mass data were acquired using the Thermo Scientific Xcalibur 4.1 software (Thermo Fisher Scientific, Waltham, MA, USA). The raw data were processed using MZmine 2.5.3 [20] and subsequently analyzed for qualitative identification with the in-house developed CNPs-MFSA application [21]. The detailed parameters used for data processing have been described in the literature [21]. Furthermore, to perform a detailed structural analysis of the detected daphnane diterpenoids, additional data processing was conducted using the Thermo Scientific FreeStyle 1.6 software. The elemental compositions of the detected peaks were calculated within a mass tolerance of ±5 ppm. The identified daphnane diterpenoids were further examined using the CAS SciFinder Discovery Platform (Chemical Abstracts Service, Columbus, OH, USA) to determine whether they corresponded to any known compounds.

## 4. Conclusions

In the present study, phytochemical analysis using UHPLC-Q-Exactive-Orbitrap MS led to the identification of 30 daphnane diterpenoids, including 12 previously unreported compounds, from the flower buds and blooming flowers of *D. odora*. Moreover, a novel daphnane diterpenoid, daphneodorin I (**16**), was successfully obtained via LC–MS-guided isolation, and its structure was elucidated using extensive physicochemical and spectroscopic analyses. Although daphnane diterpenoids have been previously isolated from various parts of *D. odora*, this is the first study to demonstrate their presence in flowers. Notably, the flower buds were found to contain a higher abundance of daphnane diterpenoids than the flowers. Furthermore, this study provides the first evidence of the occurrence of daphnane diterpenoids bearing a *Z*-configured phenolic acyl moiety in Thymelaeaceae plants [32]. Given the extremely low yield of daphneodorin I (**16**) isolated from the flower buds of *D. odora*, it is likely that the phenolic acyl moieties in this species predominantly exist in the E-configuration. The formation of *Z*-isomers may result from the photoinduced geometric isomerization of the corresponding *E*-isomers upon exposure to ultraviolet radiation in plant tissues [33,34]. Because the *Z*-isomer adopts a three-dimensional structure distinct from that of the *E*-isomer, such configurational differences may influence intermolecular interactions with biological targets and, consequently, affect bioactivity [32,35]. Collectively, these findings highlight the flower buds of *D. odora* as a rich source of structurally diverse daphnane diterpenoids and underscore their potential for further chemical and biological investigations.

## Figures and Tables

**Table 2:** ^1^H-NMR (500 MHz) and ^13^C-NMR (125 MHz) spectroscopic data for compound **16** (CDCl_3_).

No.	*δ*_H_ (*J* in Hz)	_C_, Type		No.	*δ*_H_ (*J* in Hz)	_C_, Type	
1	7.57 (1H, m)	161.3	CH	1′		116.6	C
2		136.6	C	2′	5.56 (1H, d, 15.4)	122.3	CH
3		209.6	C	3′	6.57 (1H, dd, 15.4, 10.9)	134.9	CH
4		72.1	C	4′	5.99 (1H, dd, 15.2, 10.9)	128.6	CH
5	4.27 (1H, s)	74.1	CH	5′	5.80 (1H, dt, 15.2, 7.2)	139.2	CH
6		58.6	C	6′	2.06 (2H, q, 7.2)	32.6	CH_2_
7	3.23 (1H, s)	66.3	CH	7′	1.35 (2H, quin, 7.2)	28.7	CH_2_
8	2.88 (1H, d, 2.5)	35.8	CH	8′	1.25 (2H, m)	31.3	CH_2_
9		77.9	C	9′	1.28 (2H, m)	22.5	CH_2_
10	3.73 (1H, t, 2.6)	47.8	CH	10′	0.86 (3H, t, 7.0)	14.0	CH_3_
11	2.31 (1H, q, 7.4)	44.5	CH	1″		165.9	C
12	4.91 (1H, s)	79.1	CH	2″	5.78 (1H, d, 12.2)	119.4	CH
13		83.7	C	3″	7.05 (1H, d, 12.2)	144.6	CH
14	4.23(1H, d, 2.5)	79.9	CH	4″		127.7	C
15	-	142.7	C	5″	7.15 (1H, d, 8.4)	130.7	CH
16	4.74 (1H, br s)	113.7	CH_2_	6″	6.89 (1H, d, 8.4)	115.0	CH
	4.92 (1H, br s)			7″		157.2	C
17	1.69 (3H, br s)	18.8	CH_3_	8″	6.89 (1H, d, 8.4)	115.0	CH
18	1.28 (3H, d, 7.2)	18.4	CH_3_	9″	7.15 (1H, d, 8.4)	130.7	CH
19	1.78 (3H, m)	9.8	CH_3_				
20	3.36 (1H, d, 12.6)	68.2	CH_2_				
	4.40 (1H, d, 12.6)						

## Data Availability

All new research data were presented in this contribution.

## Supplementary Materials

The following supporting information can be downloaded at https://www.mdpi.com/article/10.3390/plants14172616/s1, Table S1: Comparison of peak areas (positive ion mode) for 30 daphnane diterpenoids identified in the flower buds and flowers of *D. odora*; Figures S1–S3: HCD product ion spectra of compounds **6**, **12**, and **28**; Figure S4: Extracted ion chromatograms (XICs) in positive ion mode for compounds **7**, **8**, **10**, **14**–**18**, **23**, and **24**; Figures S5–S11: HCD product ion spectra of compounds **7**, **8**, **10**, **14**–**18**, **23**, and **24**; Figures S12–S18: 1D and 2D NMR spectra of daphneodorin I (**16**); Figure S19: UV spectrum of daphneodorin I (**16**); Figure S20: ECD spectrum of daphneodorin I (**16**); Figure S21: HRESIMS data of daphneodorin I (**16**); Figure S22: IR spectrum of daphneodorin I (**16**).
