# Supplementary material for: Identification of Daphnane Diterpenoids from Flower Buds and Blooming Flowers of Daphne odora Using UHPLC-Q-Exactive-Orbitrap MS
Source: Plants (Basel). 2025 Aug 22;14(17):2616. doi: 10.3390/plants14172616 (PMC12430396; doi:10.3390/plants14172616)
Supplement: Supplementary file 1 [file plants-14-02616-s001.zip › plants-3825569-supplementary.pdf]

Supporting Information

Identification of daphnane diterpenoids from flower buds and flowers of Daphne odora using UHPLC-Q-Exactive-Orbitrap MS

Kouharu Otsuki*, Kousei Miyamoto, Mami Goto, Mi Zhang, Takashi Kikuchi, Wei Li*

Faculty of Pharmaceutical Sciences, Toho University, Miyama 2-2-1, Funabashi, Chiba 274-8510, Japan

Contents

Table S1. Comparison of peak areas (positive ion mode) for 30 daphnane diterpenoids identified in the flower buds and flowers of *D. odora* S3
Figure S1. HCD product ion spectra of compound **6**: (A) obtained using $[M + H]^+$ ion as the precursor ion (positive ion mode, NCE: 15–20 eV) and (B) obtained using $[M - H]^-$ ion as the precursor ion (negative ion mode, NCE: 10 eV) S4
Figure S2. HCD product ion spectra of compound **12**: (A) obtained using $[M + H]^+$ ion as the precursor ion (positive ion mode, NCE: 15–20 eV) and (B) obtained using $[M - H]^-$ ion as the precursor ion (negative ion mode, NCE: 10 eV) S5
Figure S3. HCD product ion spectra of compound **28**: (A) obtained using $[M + H]^+$ ion as the precursor ion (positive ion mode, NCE: 15–20 eV) and (B) obtained using $[M + HCOO]^-$ ion as the precursor ion (negative ion mode, NCE: 10 eV).. S6
Figure S4. Extracted ion chromatograms (XICs) in positive ion mode for compounds **7**, **8**, **10**, **14–18**, **23**, and **24**.. S7
Figure S5. HCD product ion spectra of compounds **7**, **10**, and **13** obtained using $[M + H]^+$ ion as the precursor ion (positive ion mode, NCE: 15–20 eV) S8
Figure S6. HCD product ion spectra of compounds **7**, **10**, and **13** obtained using $[M - H]^-$ ion as the precursor ion (negative ion mode, NCE: 10 eV) S9
Figure S7. HCD product ion spectra of compounds **8**, **14**, and **15** obtained using $[M + H]^+$ ion as the precursor ion (positive ion mode, NCE: 15–20 eV) S10
Figure S8. HCD product ion spectra of compounds **8**, **14**, and **15** obtained using $[M - H]^-$ ion as the precursor ion (negative ion mode, NCE: 10 eV) S11
Figure S9. HCD product ion spectra of compounds **16** and **18**: (A) obtained using $[M + H]^+$ ion as the precursor ion (positive ion mode, NCE: 15–20 eV) and (B) obtained using $[M - H]^-$ ion as the precursor ion (negative ion mode, NCE: 10 eV) S12
Figure S10. HCD product ion spectra of compounds **17** and **20**: (A) obtained using $[M + H]^+$ ion as the precursor ion (positive ion mode, NCE: 15–20 eV) and (B) obtained using $[M - H]^-$ ion as the precursor ion (negative ion mode, NCE: 10 eV) S13
Figure S11. HCD product ion spectra of compounds **23** and **24**: (A) obtained using $[M + H]^+$ ion as the precursor ion (positive ion mode, NCE: 15–20 eV) and (B) obtained using $[M + HCOO]^-$ ion as the precursor ion (negative ion mode, NCE: 10 eV) S14
Figure S12–S18. 1D and 2D NMR spectra of daphneodorin I (**16**).. S15
Figure S19. UV spectrum of daphneodorin I (**16**) S19
Figure S20. ECD spectrum of daphneodorin I (**16**) S19
Figure S21. HRESI-MS data of daphneodorin I (**16**) S20
Figure S22. IR spectrum of daphneodorin I (**16**) S21

Table S1. Comparison of peak areas (positive ion mode) for 30 daphnane diterpenoids identified in the flower buds and flowers of *D. odora*.

No.	Flower buds				Flowers			
	Peak area[a]			RSD (%)	Peak area[a,b]			RSD (%)
**1**	662768	±	61016	9.2	N.D.			
**2**	1113632	±	5874	0.5	N.D.			
**3**	803293	±	9949	1.2	114071	±	5277	4.6
**4**	1985848	±	35242	1.8	N.D.			
**5**	5364719	±	87142	1.6	145593	±	363	0.2
**6**	1460430	±	16316	1.1	N.D.			
**7**	7882065	±	112102	1.4	N.D.			
**8**	6194989	±	97134	1.6	61703	±	3593	5.8
**9**	1806078	±	39619	2.2	95326	±	4145	4.3
**10**	1670586	±	55770	3.3	49191	±	831	1.7
**11**	1162260	±	30037	2.6	74292	±	4576	6.2
**12**	938143	±	28320	3.0	N.D.			
**13**	70657160	±	684222	1.0	235222	±	1359	0.6
**14**	1824855	±	24861	1.4	54595	±	4910	9.0
**15**	76974154	±	2075848	2.7	292783	±	11451	3.9
**16**	4729804	±	76913	1.6	622627	±	15234	2.4
**17**	3907959	±	103102	2.6	575106	±	10972	1.9
**18**	44514020	±	1037087	2.3	1785483	±	23480	1.3
**19**	2266592	±	46594	2.1	N.D.			
**20**	46974393	±	739904	1.6	2266458	±	50990	2.2
**21**	133270	±	11592	8.7	N.D.			
**22**	63142673	±	1375124	2.2	173117	±	13251	7.7
**23**	1070029	±	28063	2.6	27659	±	795	2.9
**24**	47825012	±	753220	1.6	165491	±	5040	3.0
**25**	41801494	±	390424	0.9	1682661	±	42118	2.5
**26**	35849439	±	774492	2.2	1355156	±	33026	2.4
**27**	5081237	±	85687	1.7	N.D.			
**28**	5459196	±	212180	3.9	N.D.			
**29**	2557542	±	36179	1.4	N.D.			
**30**	5785802	±	100238	1.7	69159	±	4999	7.2

[a]Mean ± SD (n = 3). [b]N.D. = Not Detected.

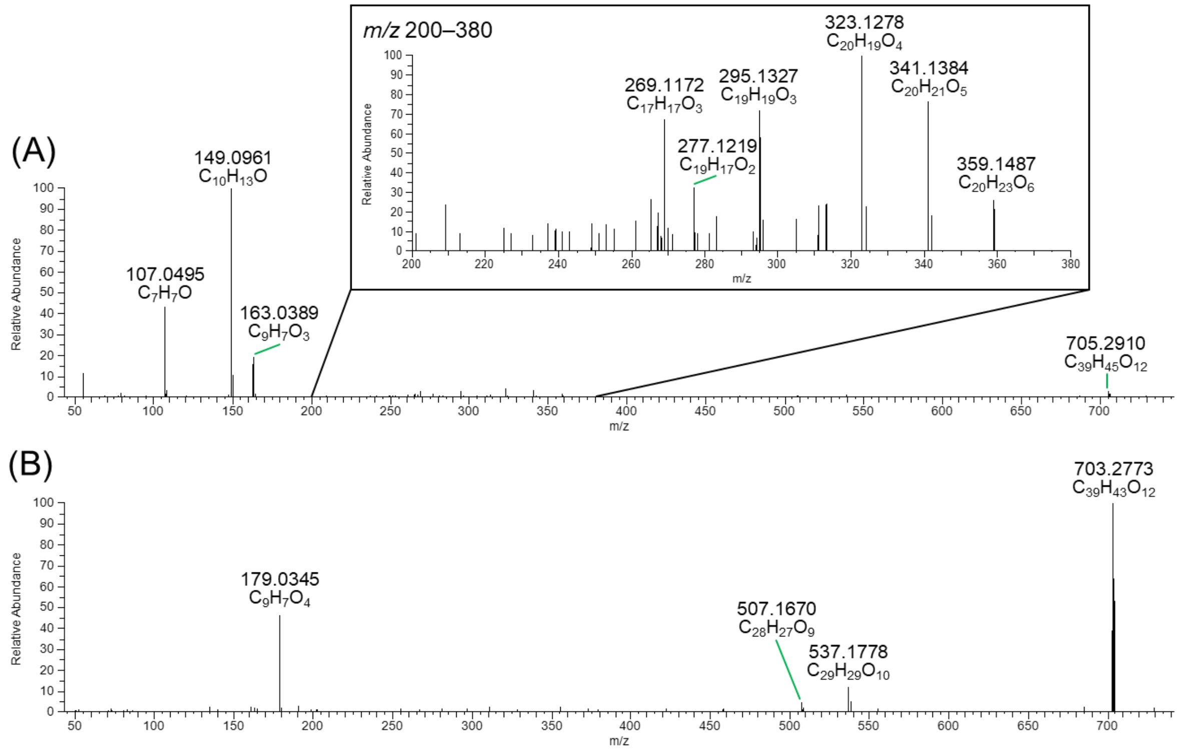

Figure S1. HCD product ion spectra of compound **6**: (A) obtained using $[M + H]^+$ ion as the precursor ion (positive ion mode, NCE: 15–20 eV) and (B) obtained using $[M - H]^-$ ion as the precursor ion (negative ion mode, NCE: 10 eV).

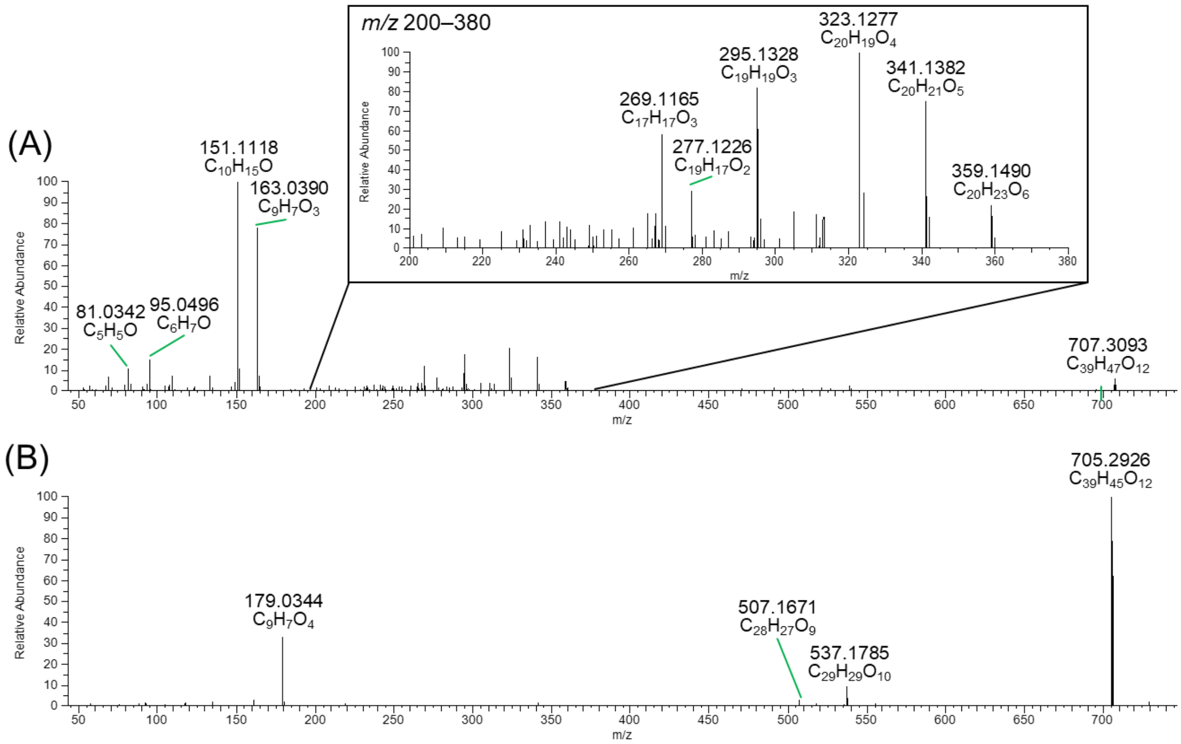

Figure S2. HCD product ion spectra of compound **12**: (A) obtained using $[M + H]^+$ ion as the precursor ion (positive ion mode, NCE: 15–20 eV) and (B) obtained using $[M - H]^-$ ion as the precursor ion (negative ion mode, NCE: 10 eV).

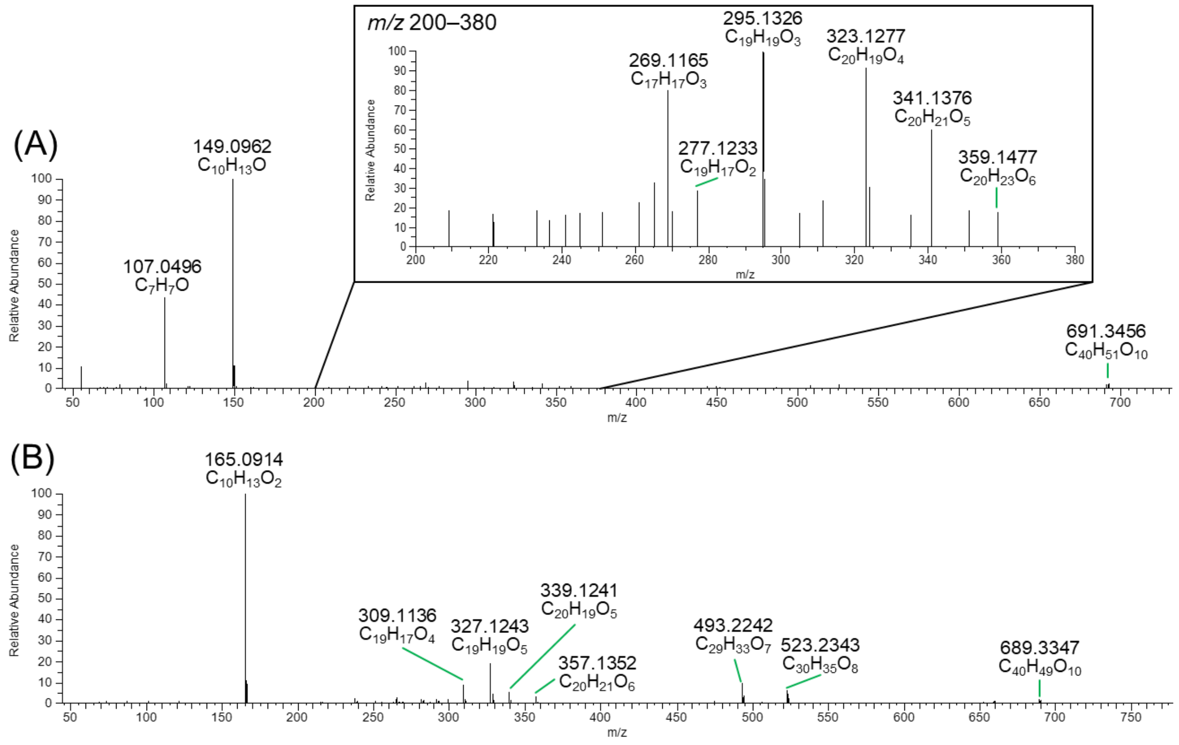

Figure S3. HCD product ion spectra of compound **28**: (A) obtained using $[M + H]^+$ ion as the precursor ion (positive ion mode, NCE: 15–20 eV) and (B) obtained using $[M + HCOO]^-$ ion as the precursor ion (negative ion mode, NCE: 10 eV).

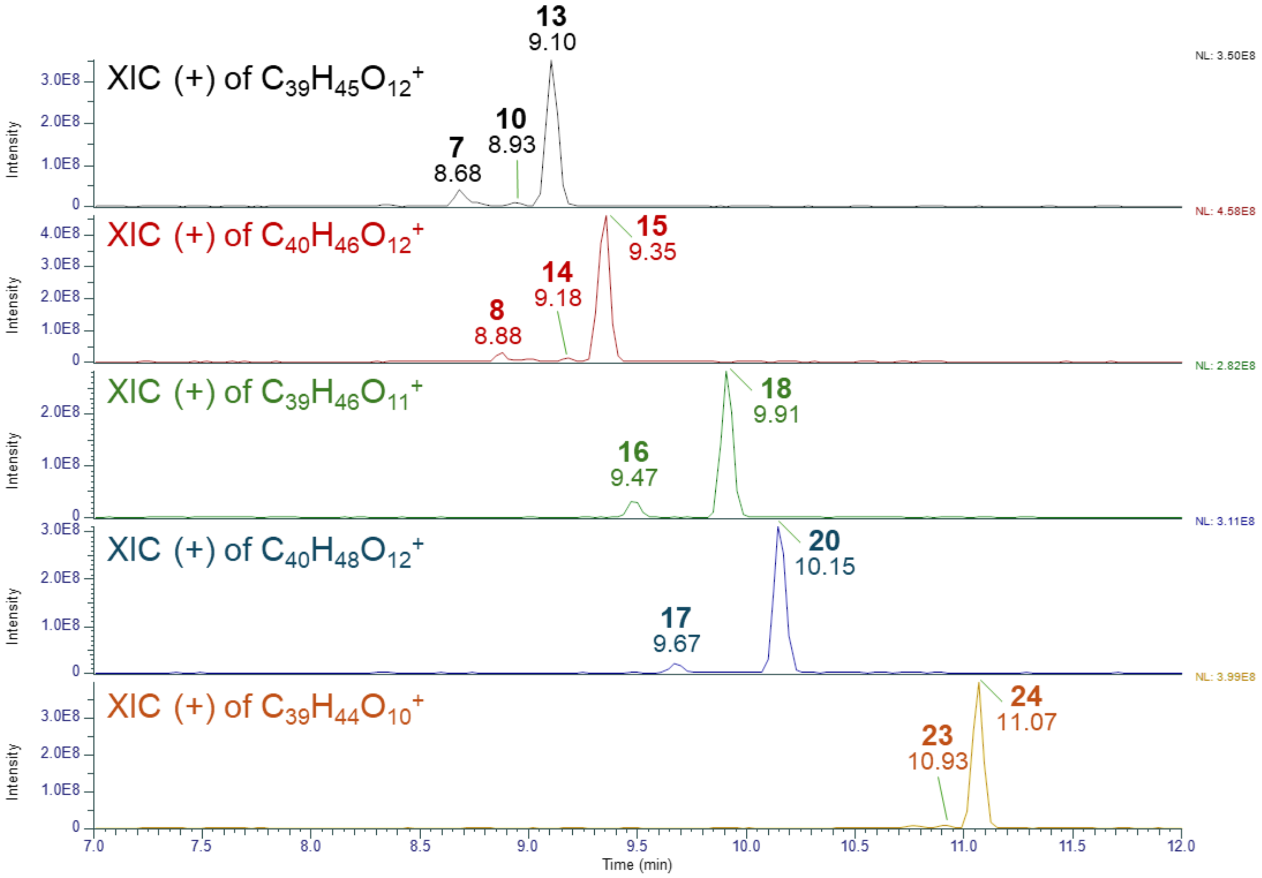

Figure S4. Extracted ion chromatograms (XICs) in positive ion mode for compounds **7**, **8**, **10**, **14**–**18**, **23**, and **24**.

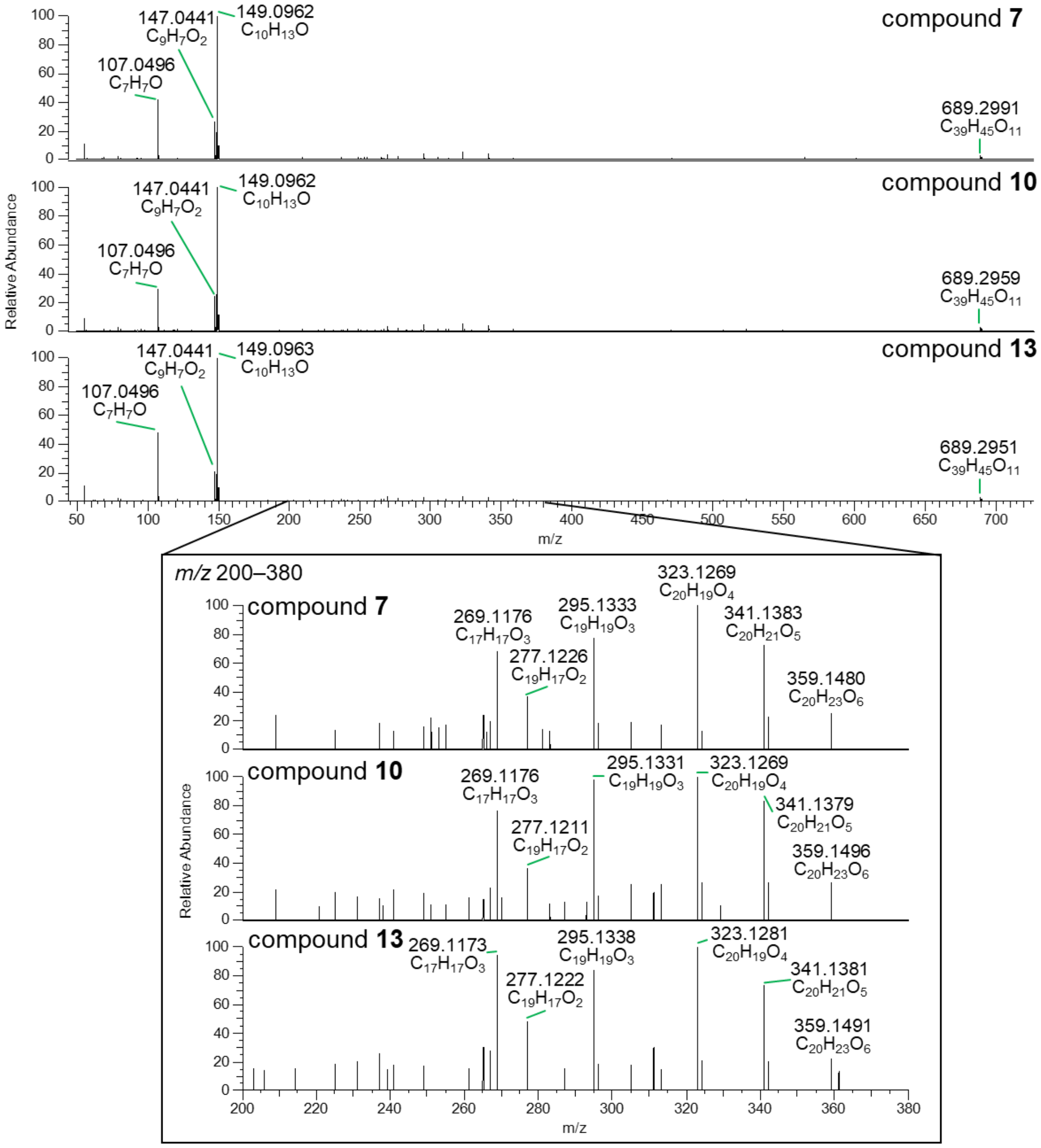

Figure S5. HCD product ion spectra of compounds **7**, **10**, and **13** obtained using $[M + H]^+$ ion as the precursor ion (positive ion mode, NCE: 15–20 eV).

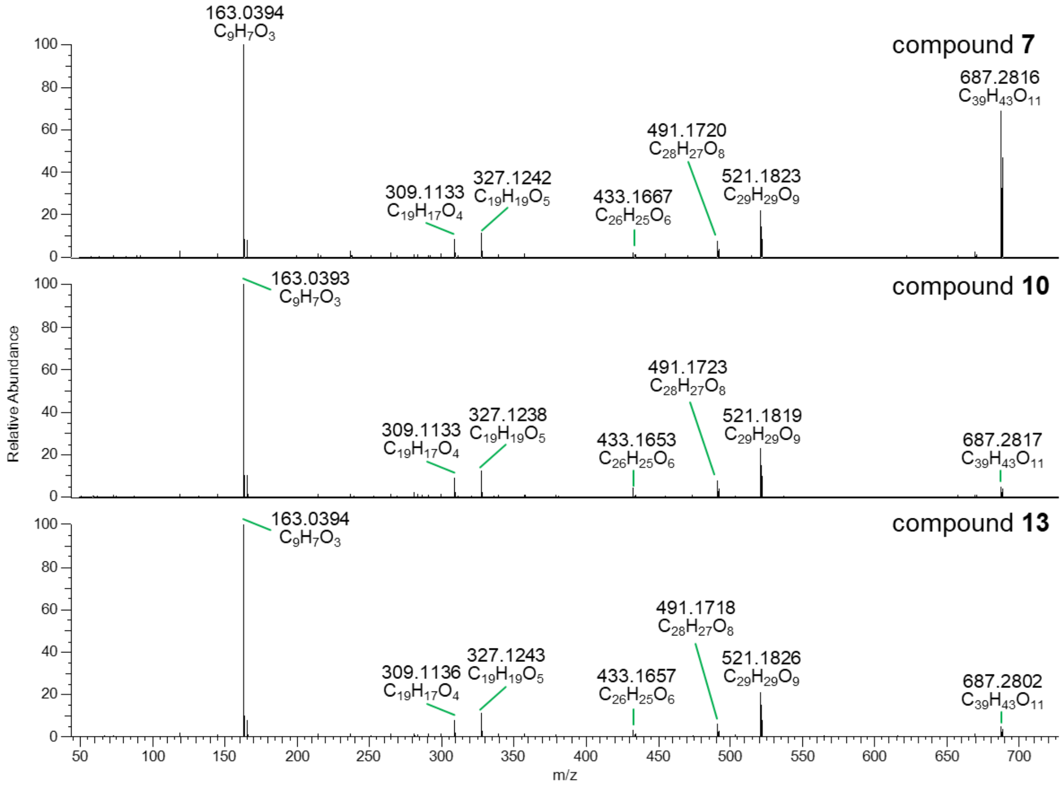

Figure S6. HCD product ion spectra of compounds **7**, **10**, and **13** obtained using $[M-H]^-$ ion as the precursor ion (negative ion mode, NCE: 10 eV).

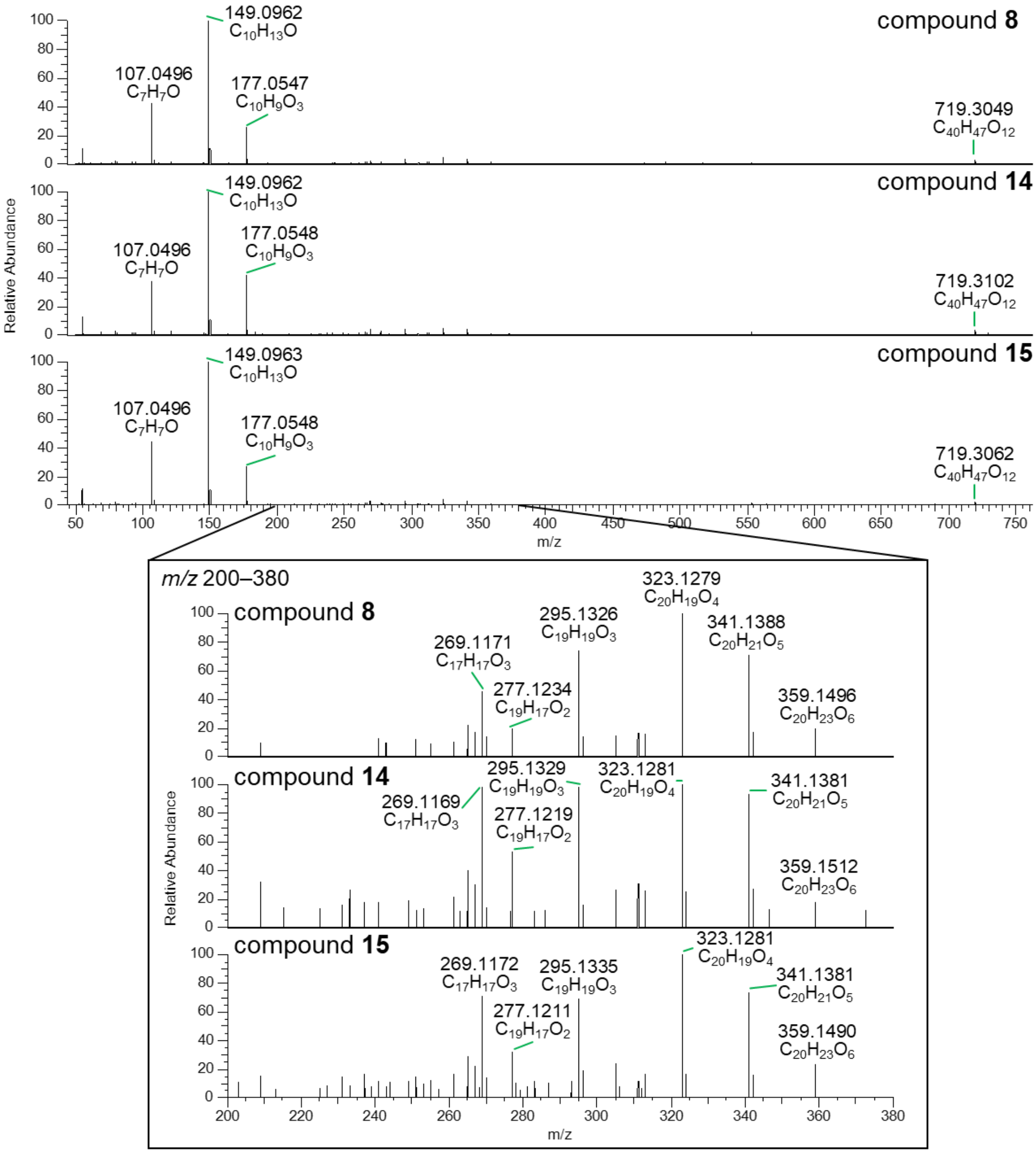

Figure S7. HCD product ion spectra of compounds **8**, **14**, and **15** obtained using $[M + H]^+$ ion as the precursor ion (positive ion mode, NCE: 15–20 eV).

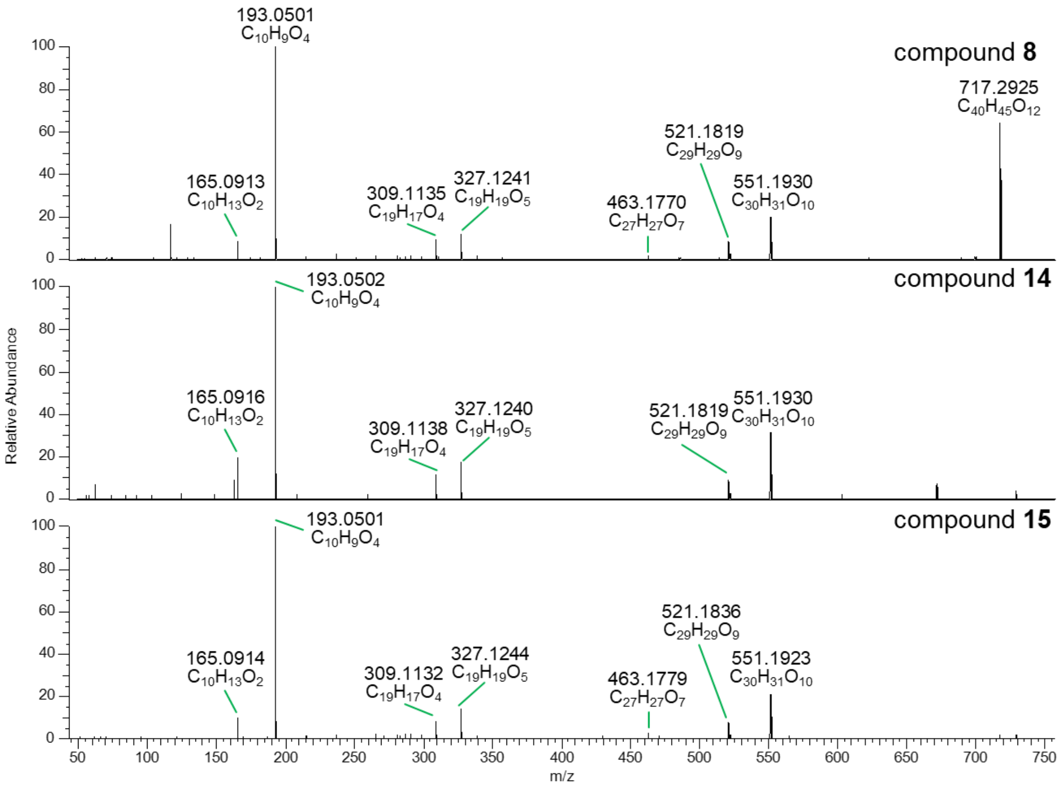

Figure S8. HCD product ion spectra of compounds **8**, **14**, and **15** obtained using $[M - H]^-$ ion as the precursor ion (negative ion mode, NCE: 10 eV).

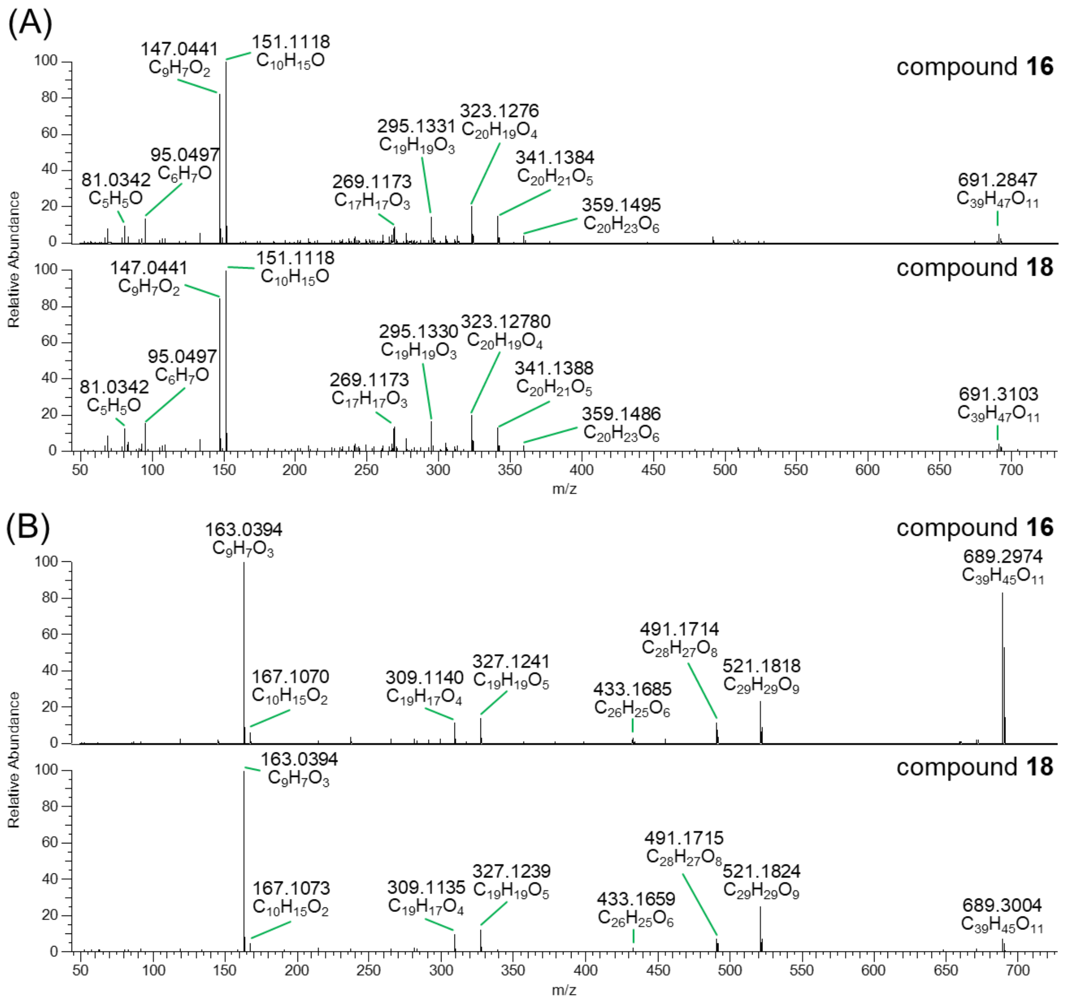

Figure S9. HCD product ion spectra of compounds **16** and **18**: (A) obtained using $[M + H]^+$ ion as the precursor ion (positive ion mode, NCE: 15–20 eV) and (B) obtained using $[M - H]^-$ ion as the precursor ion (negative ion mode, NCE: 10 eV).

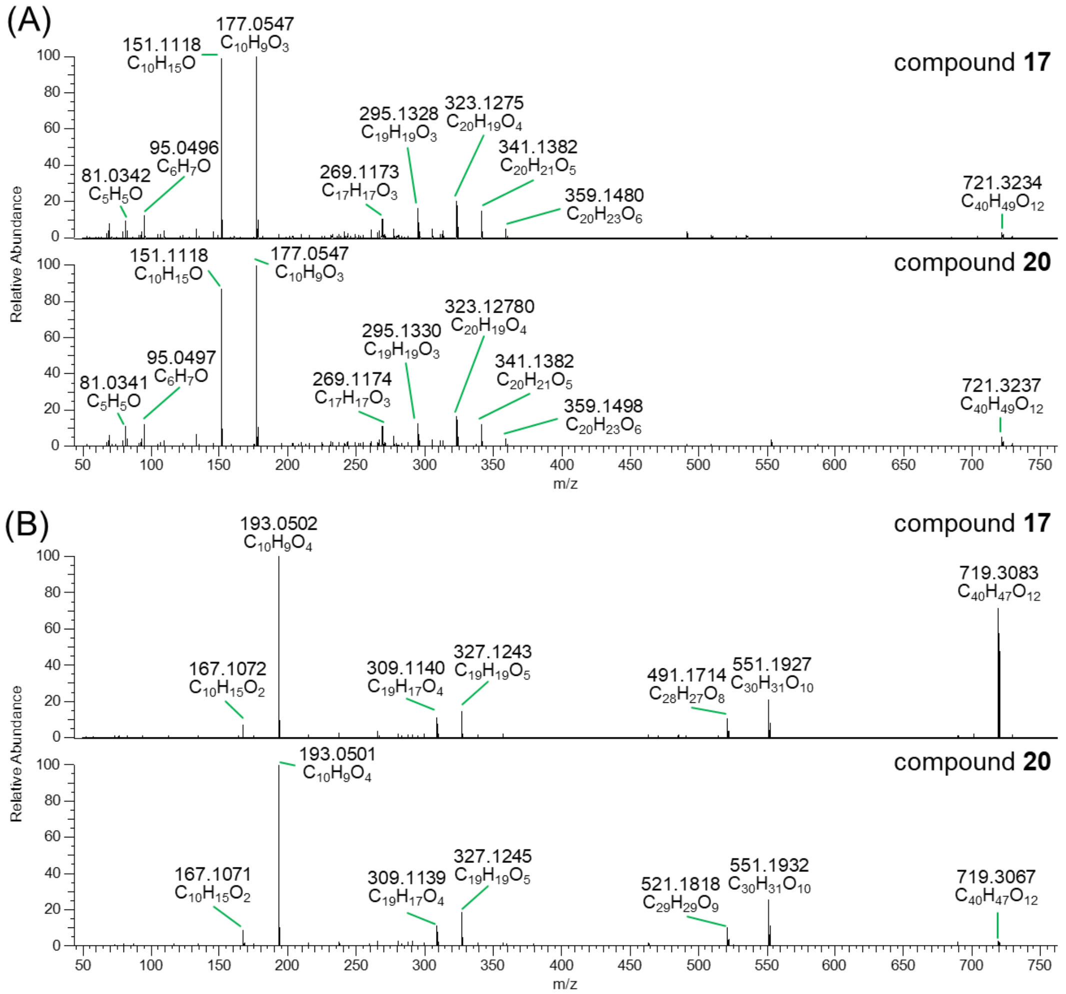

Figure S10. HCD product ion spectra of compounds **17** and **20**: (A) obtained using $[M + H]^+$ ion as the precursor ion (positive ion mode, NCE: 15–20 eV) and (B) obtained using $[M - H]^-$ ion as the precursor ion (negative ion mode, NCE: 10 eV).

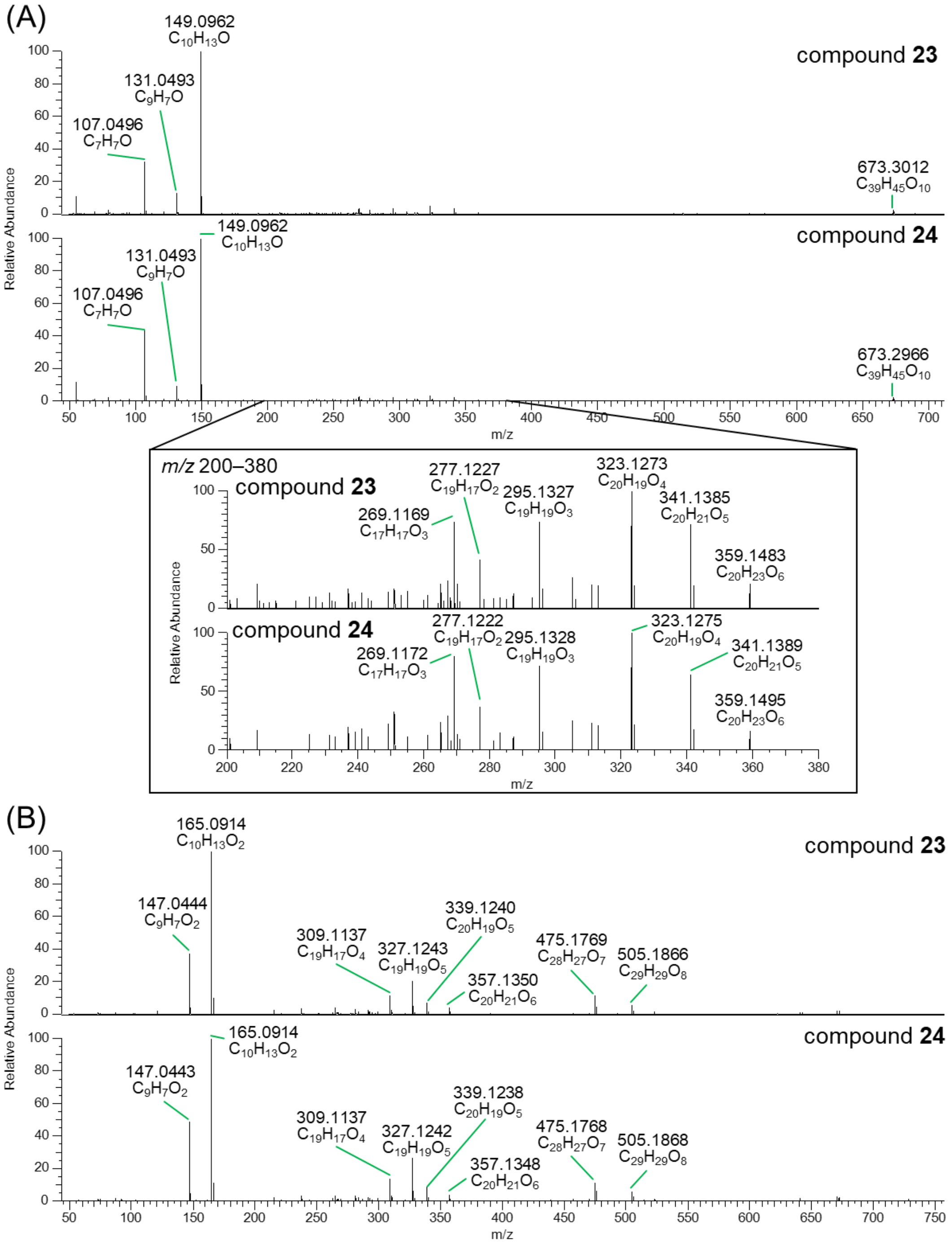

Figure S11. HCD product ion spectra of compounds **23** and **24**: (A) obtained using $[M + H]^+$ ion as the precursor ion (positive ion mode, NCE: 15–20 eV) and (B) obtained using $[M + HCOO]^-$ ion as the precursor ion (negative ion mode, NCE: 10 eV).

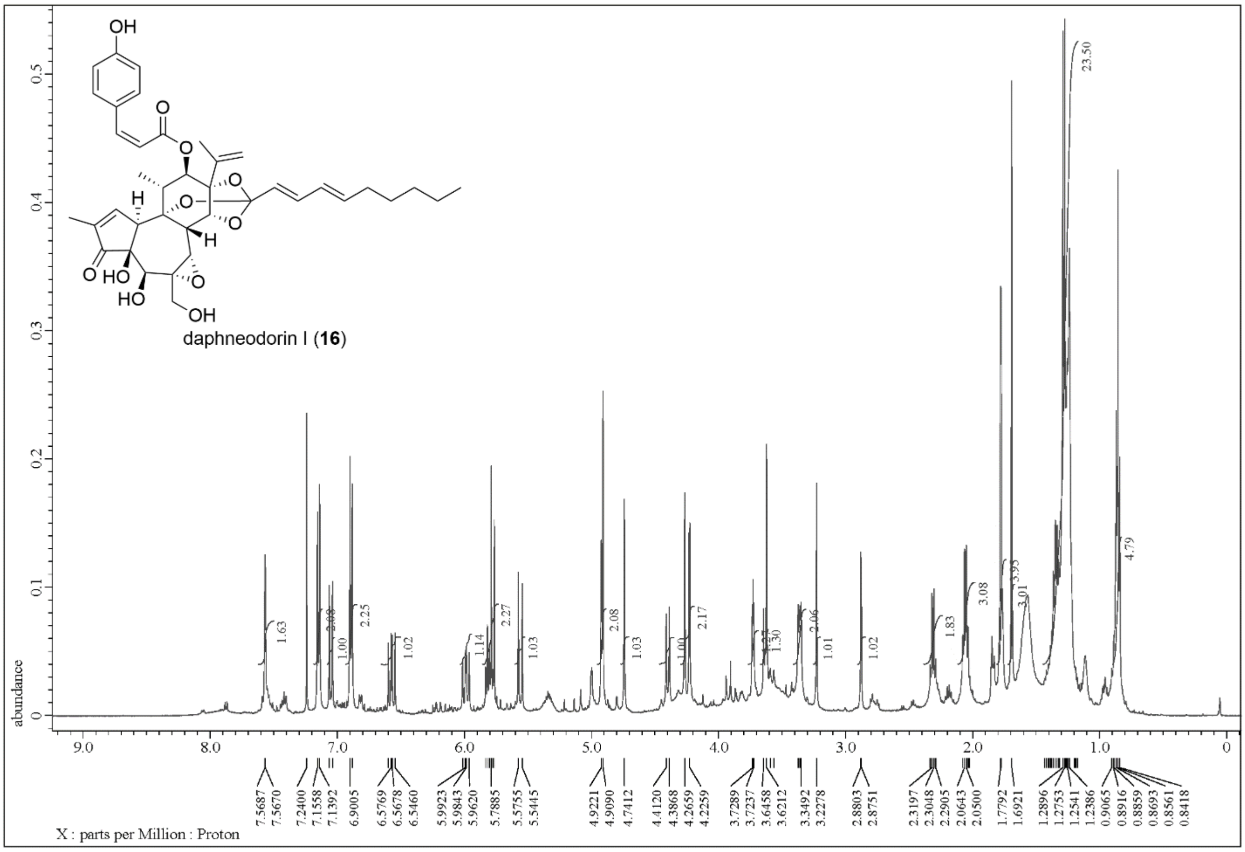

Figure S12. ^{1}H-NMR spectrum of daphneodorin I (**16**).

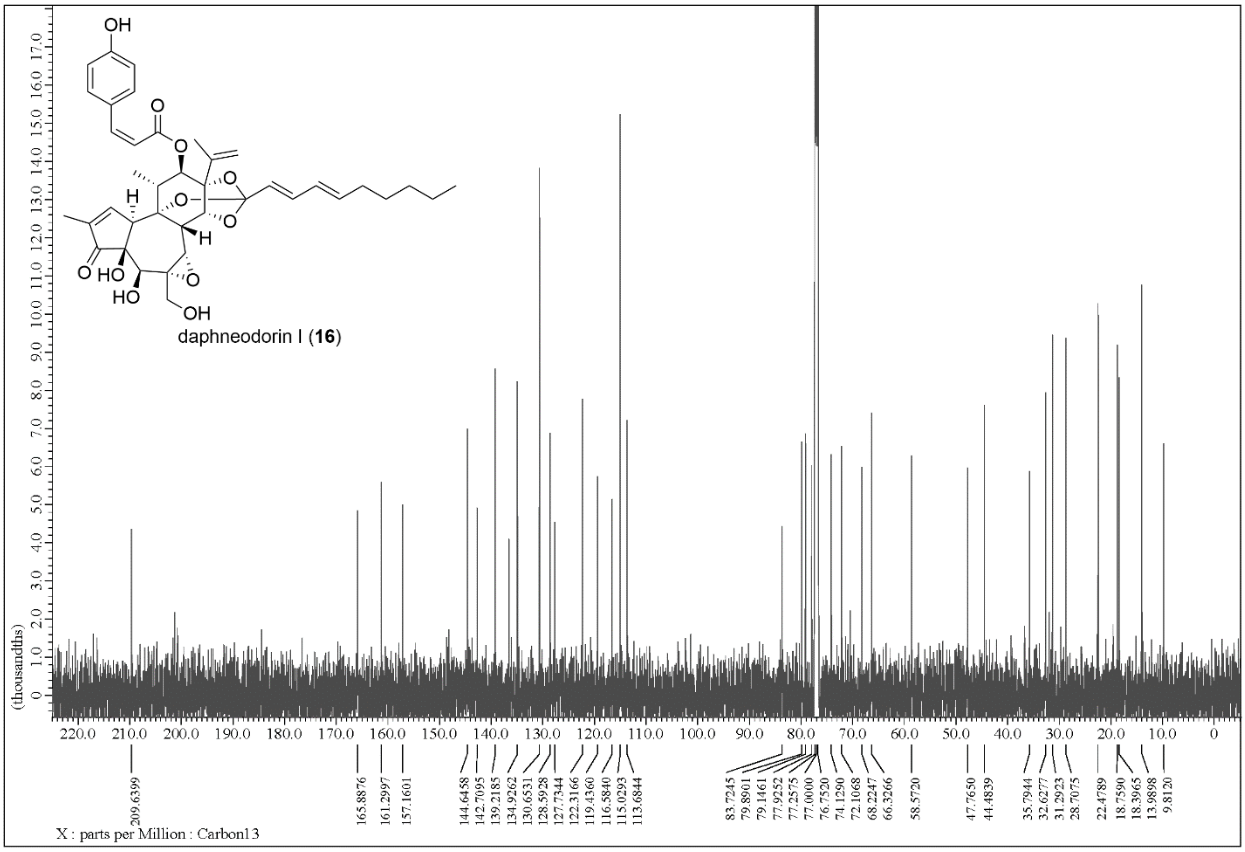

Figure S13. ^{13}C-NMR spectrum of daphneodorin I (**16**).

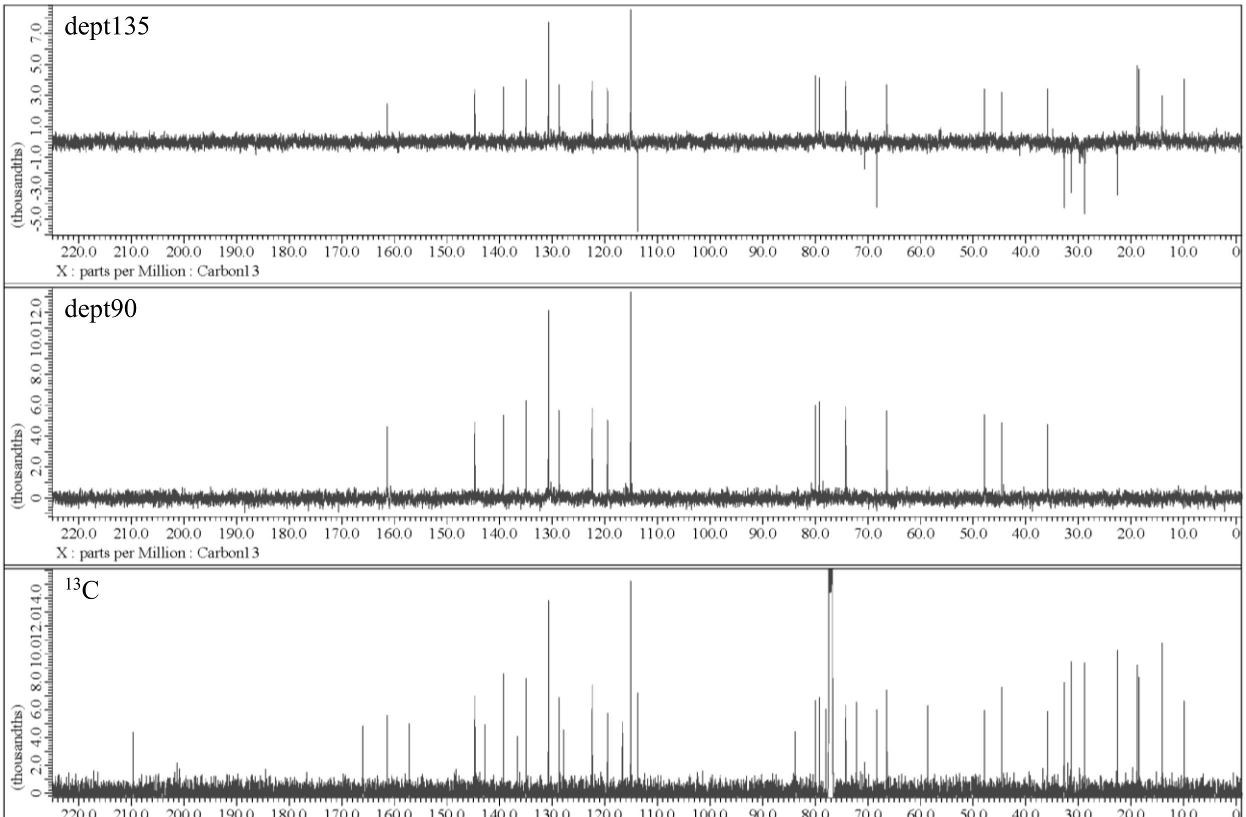

Figure S14. DEPT spectra of daphneodorin I (**16**).

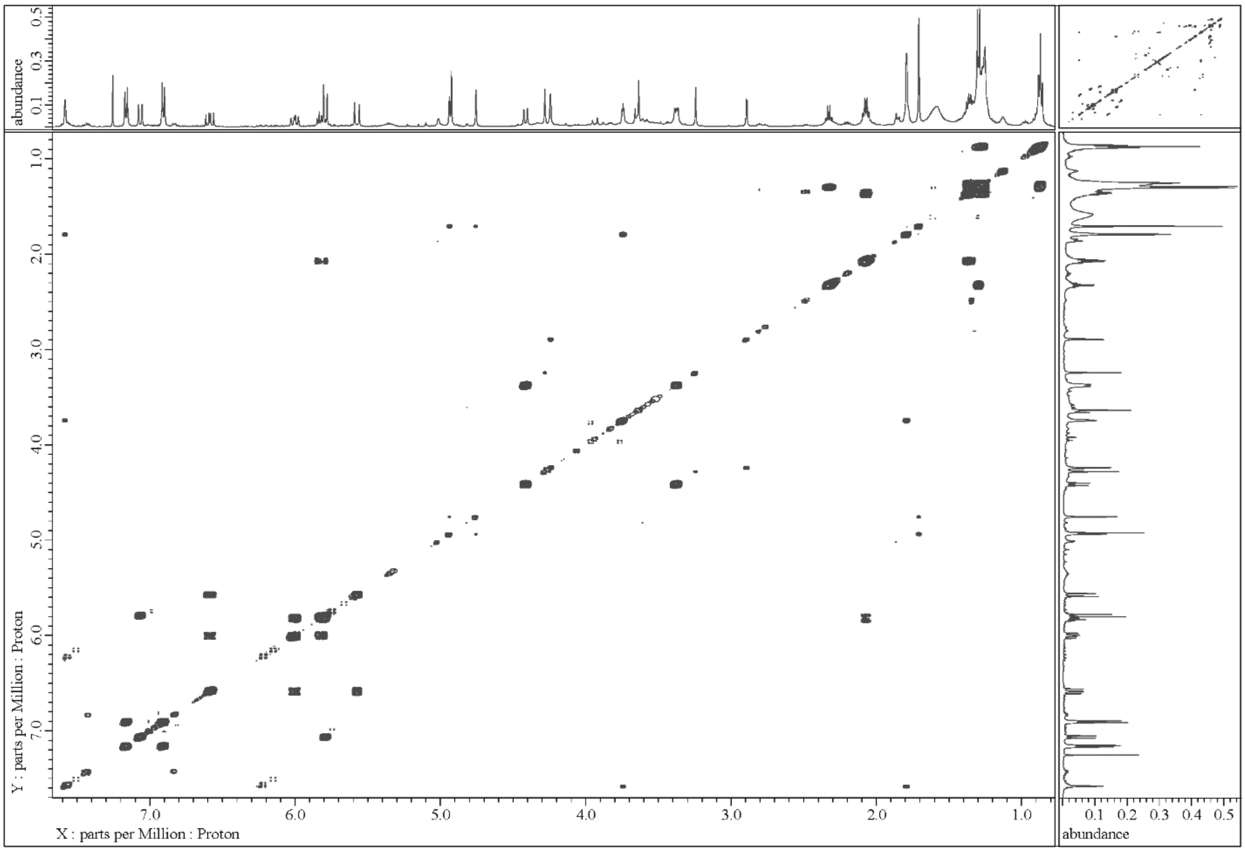

Figure S15. ^{1}H–^{1}H COSY spectrum of daphneodorin I (**16**).

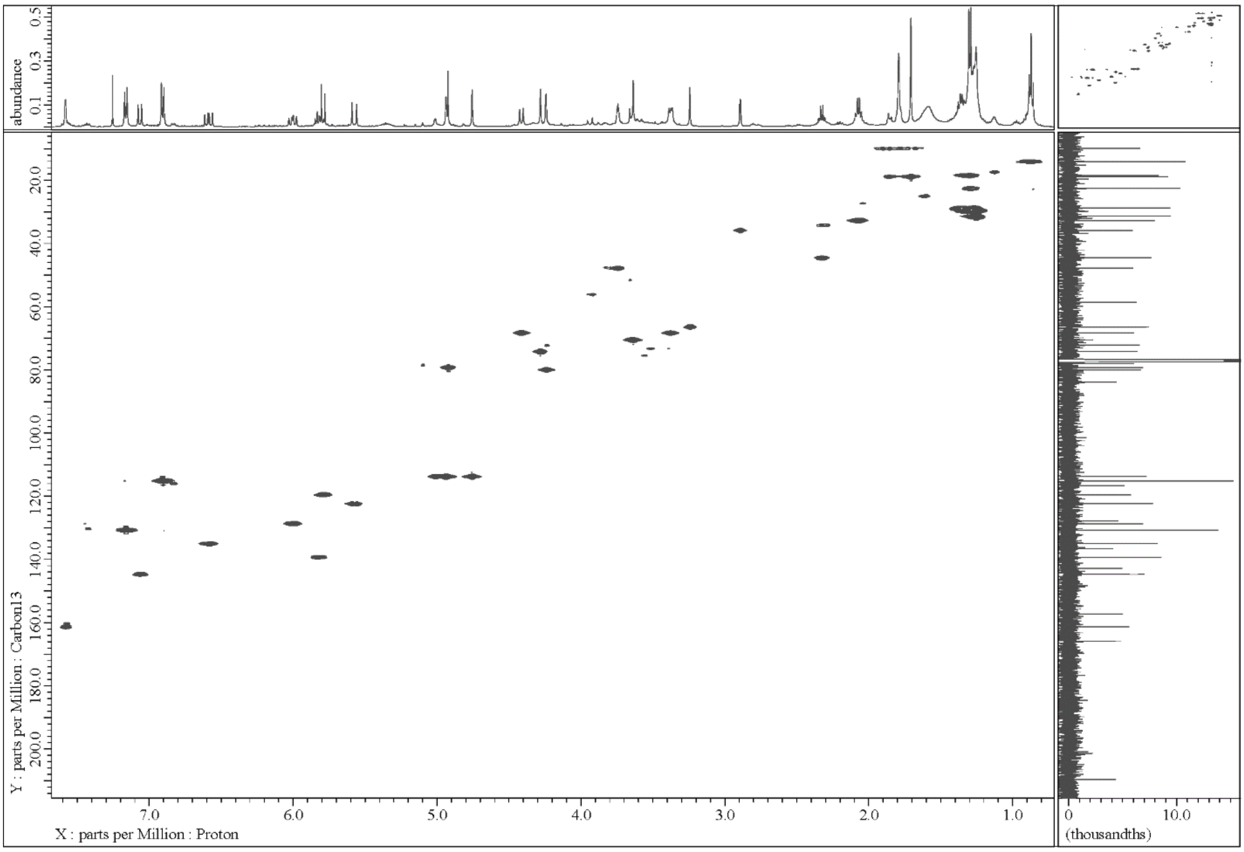

Figure S16. HSQC spectrum of daphneodorin I (**16**).

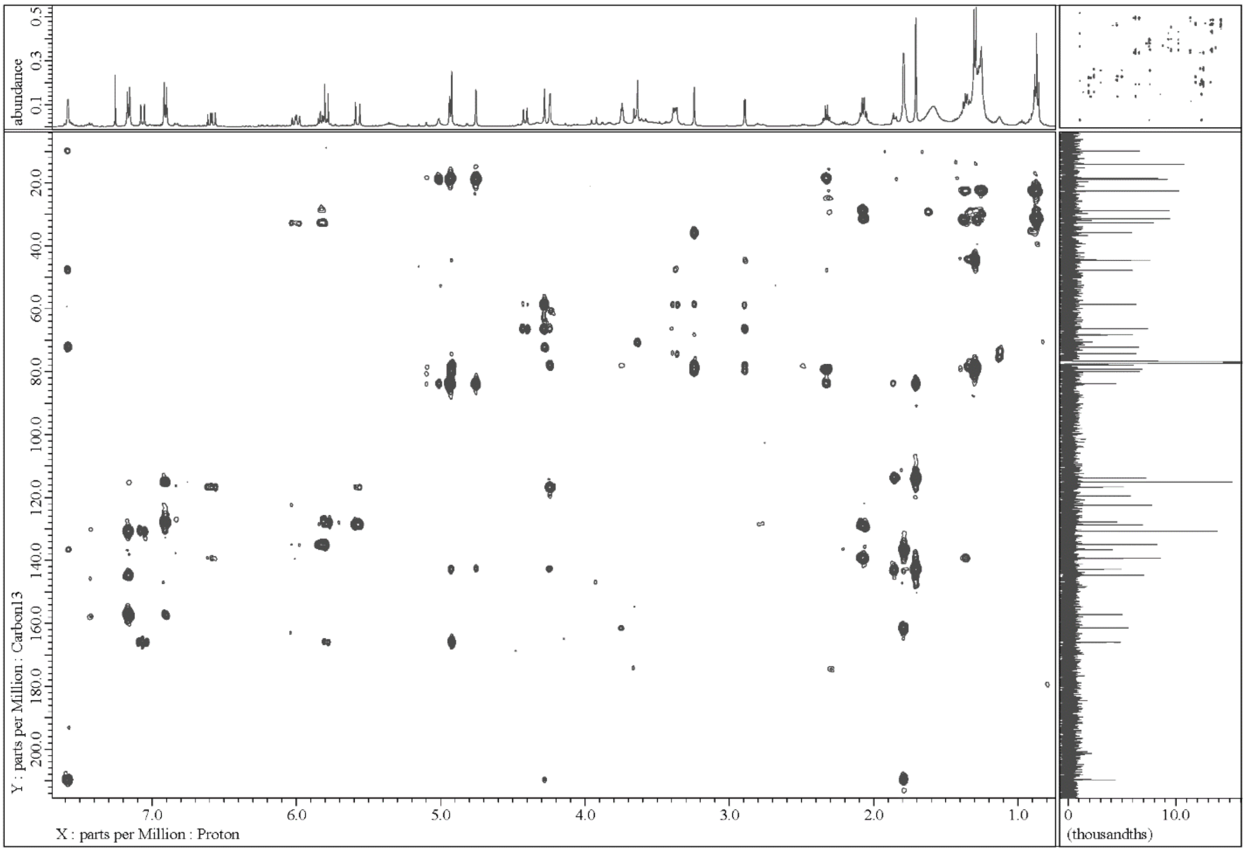

Figure S17. HMBC spectrum of daphneodorin I (**16**).

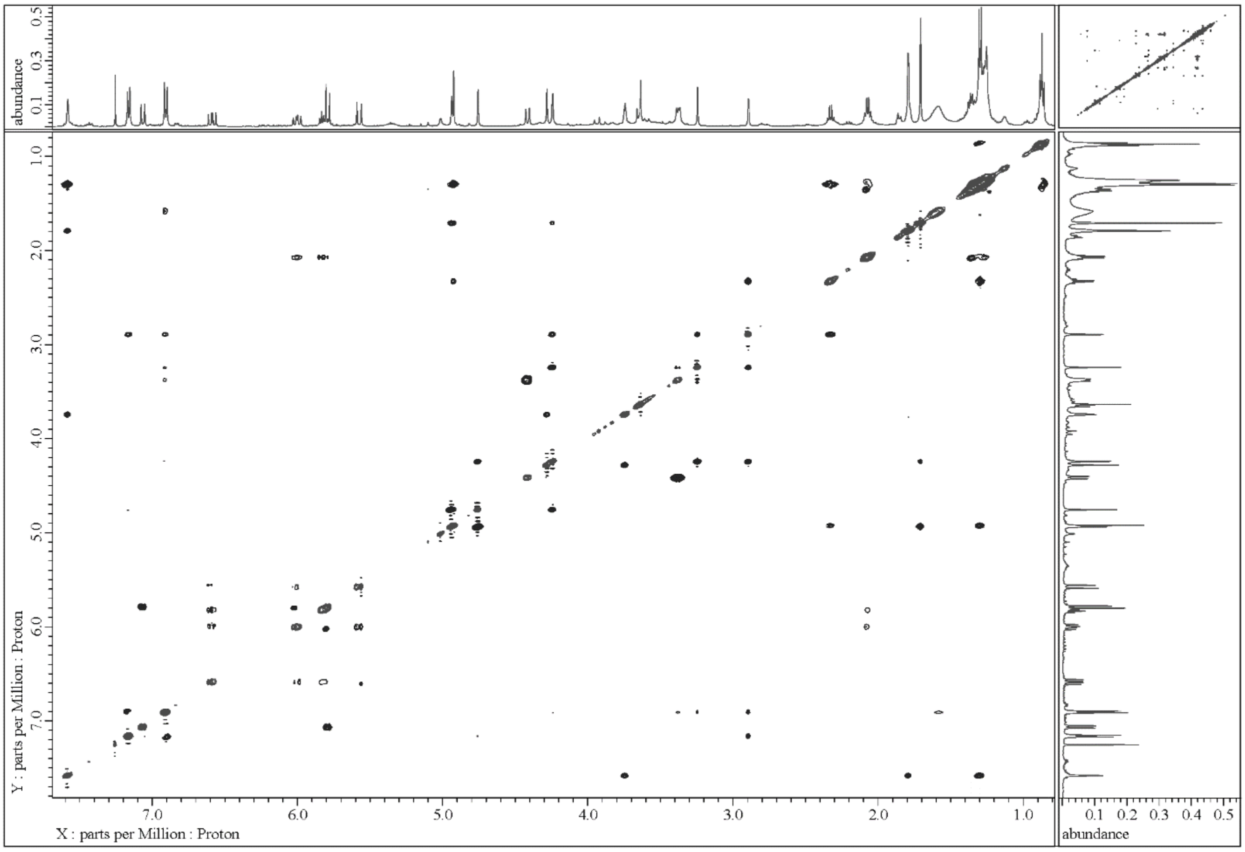

Figure S18. NOESY spectrum of daphneodorin I (**16**).

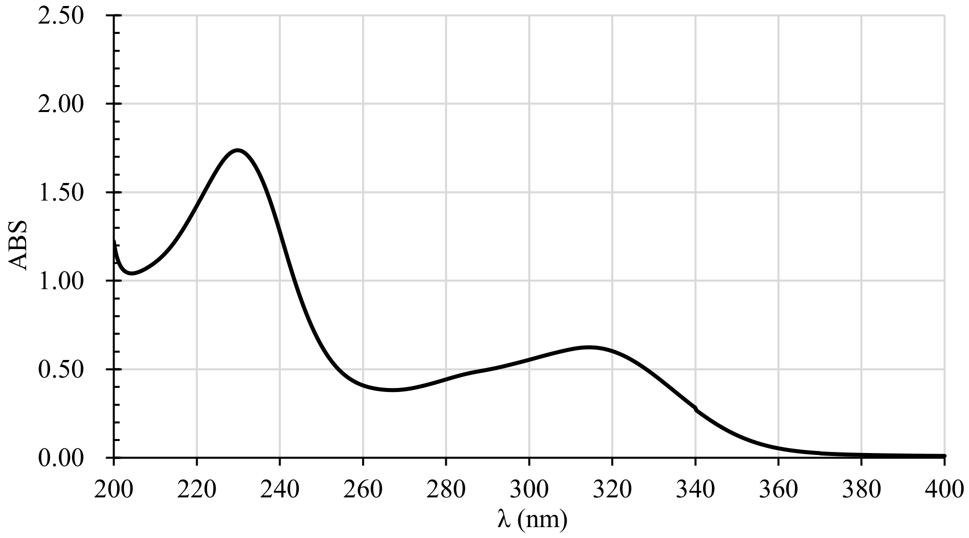

Figure S19. UV spectrum of daphneodorin I (**16**).

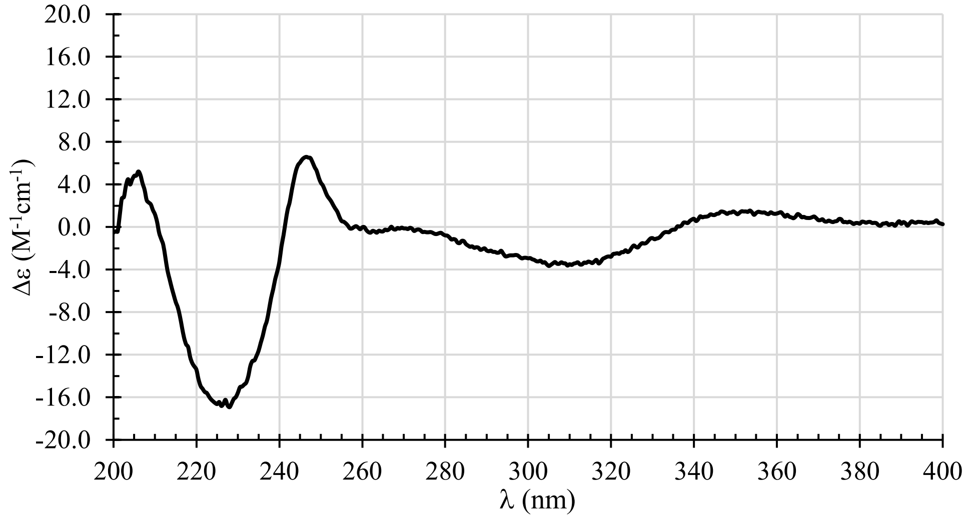

Figure S20. ECD spectrum of daphneodorin I (**16**).

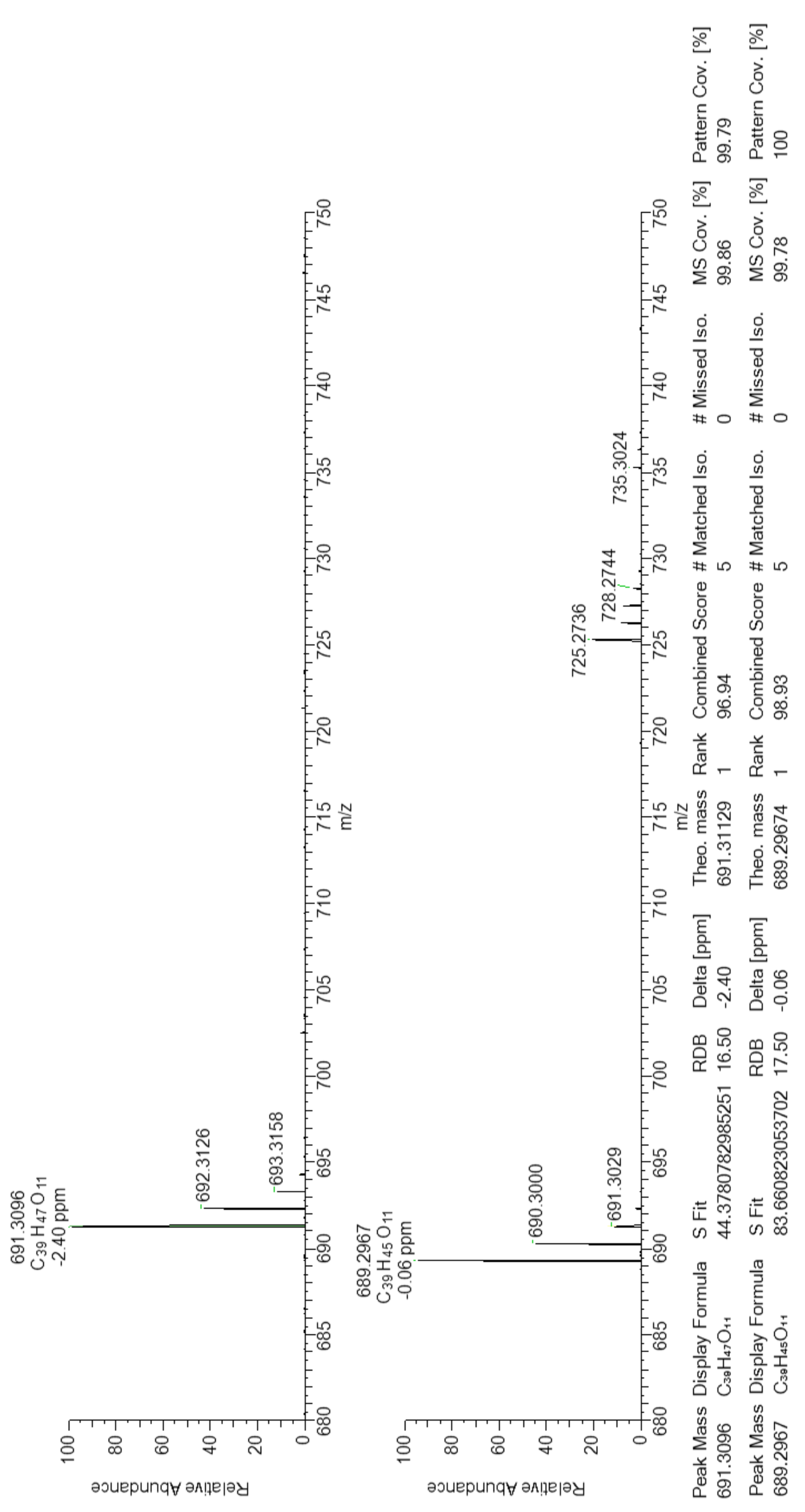

Figure S21. HRESI-MS data of daphneodorin I (**16**).

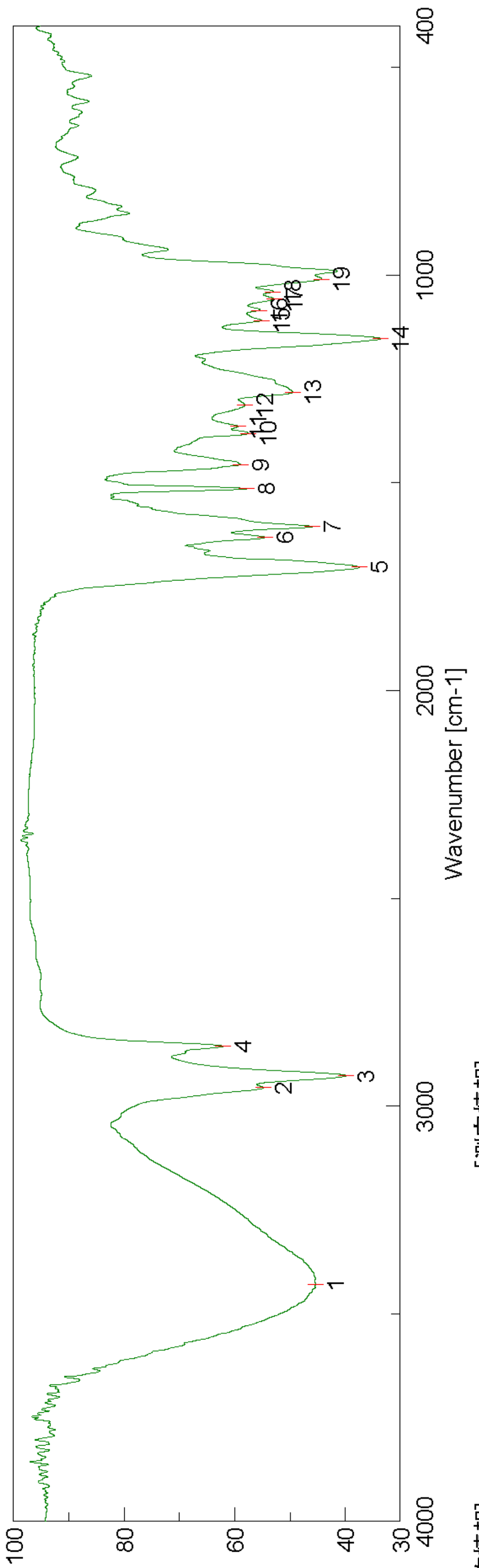

[データ情報]
作成日時　2025/06/24 20:33

データタイプ　等間隔データ
横軸　Wavenumber [cm-1]
縦軸　%T
スタート　399.193 cm-1
エンド　4000.6 cm-1
データ間隔　0.964233 cm-1
データ数　3736

[測定情報]
機種名　FT/IR-4X1typeA
シリアル番号　A060062111

[ピーク検出結果]

No.	位置	強度	No.	位置	強度	No.	位置	強度	No.	位置	強度
1	3428.81	45.2642	2	2955.38	54.6823	3	2927.41	39.8264	4	2856.06	62.0749
5	1702.84	37.3972	6	1631.48	54.5229	7	1605.45	45.8793	8	1513.85	57.7796
9	1455.99	58.8074	10	1380.78	57.4457	11	1363.43	59.2707	12	1312.32	58.1064
13	1281.47	49.4011	14	1153.22	33.5508	15	1108.87	55.1256	16	1084.76	55.5418
17	1056.8	52.8227	18	1040.41	53.0847	19	1009.55	44.2921			

Figure S22. IR spectrum of daphneodorin I (**16**).
